# Current advances in psittacine beak and feather disease: From molecular pathogenesis to integrated surveillance and global control strategies

**DOI:** 10.14202/vetworld.2026.3522-3557

**Published:** 2026-08-11

**Authors:** Hongmin Guan

**Affiliations:** 1 Department of Pet Science and Technology, Zhengzhou Construction Vocational College, Zhengzhou 451200, China.

**Keywords:** beak and feather disease virus, biosecurity, circovirus, conservation, diagnostics, molecular epidemiology, psittacine beak and feather disease, vaccination

## INTRODUCTION

Psittacine beak and feather disease (PBFD) is one of the most important viral diseases affecting members of the order *Psittaciformes*, with major implications for companion birds, commercial aviculture, wildlife rehabilitation, and the conservation of endangered parrot species [1, 2]. The etiological agent, beak and feather disease virus (BFDV), is a non-enveloped, icosahedral circovirus possessing an ambisense, circular, single-stranded DNA (ssDNA) genome of approximately 1,990–2,020 bp [3, 4]. Clinical manifestations range from peracute disease in juvenile birds to chronic feather dystrophy, beak lesions, immunosuppression, and persistent asymptomatic infection [2, 5]. BFDV is transmitted through subclinical carriers, contaminated environments, and the international live-bird trade, thereby affecting both individual animal health and population-level biosecurity [6–8].

Over recent decades, PBFD has evolved from a geographically localized disease into a globally distributed disease of considerable veterinary and conservation importance. It now represents a persistent biosecurity concern for captive breeding facilities, zoological collections, wildlife hospitals, and species-recovery programs. Since 1975, more than 19 million parrots have been transported through the legal international pet trade, creating repeated opportunities for viral dissemination between captive and wild populations [6]. The greatest conservation impact occurs in small, island-endemic, or reintroduced populations that possess limited demographic resilience and reduced immunogenetic diversity. Furthermore, climate-associated environmental stressors, habitat degradation, food scarcity, and increased aggregation around feeding or release sites may increase viral exposure and transmission. Representative examples include studies involving orange-bellied parrots and Cape parrots [9, 10], together with recent reports involving great green macaws and Spix's macaws [11, 12]. Increasing molecular detection of BFDV in non-psittacine avian species has further expanded the epidemiological scope of surveillance. However, in the absence of evidence demonstrating viral replication and onward transmission, polymerase chain reaction (PCR) positivity alone should be interpreted as evidence of exposure or passive carriage rather than true infection [13, 14]. Consequently, a comprehensive understanding of the global epidemiology, transmission dynamics, and remaining control challenges associated with BFDV is essential for strengthening conservation initiatives and improving avian biosecurity.

Despite substantial advances in molecular virology, epidemiology, and diagnostic technologies, several important knowledge gaps continue to limit the effective control of PBFD. Current evidence regarding viral evolution, recombination, environmental persistence, host susceptibility, and cross-species transmission remains fragmented, with limited integration across molecular, ecological, and conservation disciplines. Although complete-genome sequencing has improved understanding of BFDV genetic diversity, the implications of emerging genotypes and recombination events for diagnostic accuracy, antigenic variation, and vaccine effectiveness remain largely unresolved [15, 16]. Likewise, recent developments in loop-mediated isothermal amplification (LAMP), quantitative PCR (qPCR), environmental DNA surveillance, and serological platforms have substantially enhanced detection capabilities; however, standardized diagnostic protocols applicable across diverse host species, sample matrixes, and field conditions are still lacking [5, 17].

Promising vaccine platforms, including DNA vaccines, messenger RNA (mRNA) vaccines, plant-produced capsid proteins, and thermostable virus-like particles (VLPs), remain at the preclinical stage. Their protective efficacy, duration of immunity (DOI), cross-clade protection, safety in juveniles, and applicability under field conditions have not been comprehensively validated [18–20]. Considerable uncertainty also remains regarding intermittent viral shedding, environmental persistence, wildlife contact networks, and the effectiveness of biosecurity measures under field conditions [7, 8, 21–23]. Furthermore, molecular detection in non-psittacine birds cannot reliably distinguish passive viral DNA carriage from productive infection and true reservoir competence [14, 24]. Collectively, these limitations highlight the need for an integrated framework that combines molecular surveillance, ecological monitoring, genomic intelligence, and evidence-based interventions to support the sustainable global control of PBFD.

**Figure 1:**
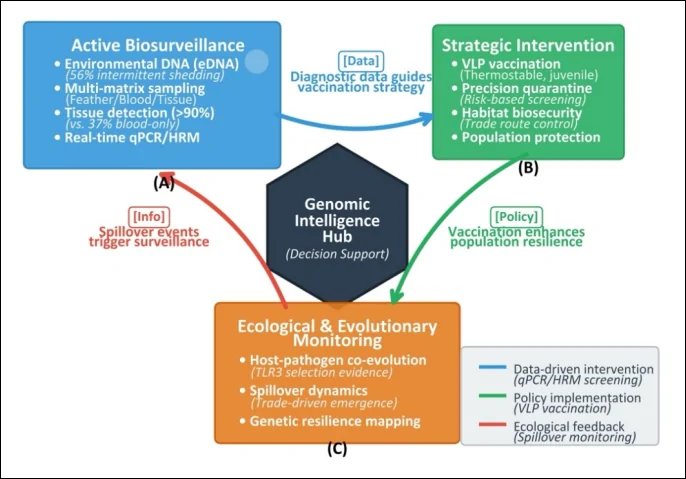
BFDV Resilience Framework. The framework is organized around three interconnected pillars coordinated through a central Genomic Intelligence Hub. (A) Active Biosurveillance integrates multi-matrix and environmental sampling to reduce the likelihood of missed intermittent shedding or tissue-restricted infection [7, 8]. (B) Strategic Intervention considers vaccine candidates as preclinical tools that should complement quarantine, exposure reduction, and biosecurity measures before field deployment [19, 23, 25]. (C) Ecological and Evolutionary Monitoring tracks host-resilience, viral recombination, and trade-associated viral movement [16, 26]. Arrows indicate continuous feedback among surveillance data, intervention strategies, and ecological monitoring.

This review synthesizes current knowledge regarding the molecular biology, pathogenesis, epidemiology, genetic evolution, diagnostic approaches, vaccine development, and control strategies of BFDV. Particular emphasis is placed on recent advances in complete-genome analysis, molecular surveillance, environmental monitoring, genomic epidemiology, and emerging vaccine technologies. In addition, this review proposes an integrated three-pillar BFDV Resilience Framework comprising active biosurveillance, strategic intervention, and ecological and evolutionary monitoring (Figure 1). The framework is complemented by a pilot BFDV Operational Risk Score that integrates viral burden, diagnostic sample matrixes, conservation status, genomic risk, trade connectivity, and host immune indicators into a tiered decision-making system. By integrating evidence across molecular, clinical, ecological, and conservation disciplines, this review aims to identify unresolved research questions, establish future priorities, and provide practical guidance for improving surveillance, biosecurity, conservation management, and the long-term global control of PBFD.

## REVIEW METHODOLOGY

A structured literature review was conducted to synthesize current knowledge on psittacine BFDV, with emphasis on viral biology, molecular epidemiology, diagnostics, host-pathogen interactions, vaccine development, conservation implications, and future disease control strategies. Electronic searches were performed using PubMed**, **Scopus**, **Web of Science**, **ScienceDirect**, **and Google Scholar to identify relevant peer-reviewed publications published between 1984 and March 2026. Additional reports, guidelines, and policy documents from recognized international organizations, including the Convention on International Trade in Endangered Species of Wild Fauna and Flora (CITES), the World Organization for Animal Health (WOAH), and the International Union for Conservation of Nature (IUCN), were consulted where relevant to wildlife health and conservation.

The search strategy combined Medical Subject Headings (MeSH), keywords, and Boolean operators, including "beak and feather disease virus", "BFDV", "psittacine beak and feather disease", "circovirus", "psittacine", "molecular epidemiology", "genomics", "diagnosis", "PCR", "high resolution melting", "LAMP", "environmental DNA", "vaccine", "virus-like particles", "conservation", "wildlife trade", "biosecurity", and "One Health".

Original research articles, review articles, surveillance studies, experimental investigations, genomic analyses, diagnostic validation studies, vaccine development reports, wildlife conservation studies, and official technical documents published in English were considered eligible. Studies unrelated to BFDV, publications lacking sufficient methodological detail, conference abstracts without full-text availability, duplicate reports, and non-scientific sources were excluded. Reference lists of eligible publications were also manually screened to identify additional relevant studies.

Information extracted from the selected literature included study objectives, host species, geographic origin, diagnostic methodology, genomic findings, epidemiological characteristics, conservation relevance, and principal outcomes. The retrieved evidence was critically evaluated and synthesized narratively to identify current advances, persistent knowledge gaps, translational challenges, and future research priorities. Because of the heterogeneity of study designs, host species, diagnostic approaches, and reported outcomes, a qualitative narrative synthesis was considered the most appropriate method of evidence integration for this review.

## VIRAL STRUCTURE AND GENOME ORGANIZATION

### Virion architecture

BFDV belongs to the genus *Circovirus* within the family *Circoviridae* [27]. The virion is a small T = 1 icosahedral particle measuring approximately 14–16 nm in diameter and lacks a lipid envelope [28, 29]. This non-enveloped architecture contributes substantially to the environmental stability of the virus, thereby complicating disease control and environmental decontamination. Conversely, its relatively simple structural organization has facilitated detailed investigations of capsid assembly, genome packaging, nuclear trafficking, and antigenic surface topology.

Current knowledge of BFDV virion structure is primarily derived from high-resolution structural analyses of the capsid protein (Cap) published in 2016 and subsequent comparisons with porcine circovirus (PCV) structural data [28, 30]. In the primary dataset, recombinant BFDV Cap formed three major structures: a 10-nm decamer resolved at 2.0 Å, a 17-nm T = 1 60-mer VLP resolved at 2.5 Å, and a 60-mer-ssDNA complex resolved at 2.3 Å. Electron microscopy further supported ssDNA-mediated stabilization of capsid assembly [30]. The same investigation demonstrated that exposure of the N-terminal arginine-rich motif/nuclear localization signal (ARM/NLS) differed between the decameric and 60-mer states and identified an internal ssDNA-binding interface. Collectively, these findings establish a mechanistic relationship among genome availability, nuclear trafficking, genome encapsidation, and capsid maturation. Comparative structural analyses further indicate that circovirus capsids share a conserved jelly-roll core but differ in loop exposure, antigenic topology, and assembly-stability characteristics, all of which are relevant to neutralization mechanisms and VLP-based vaccine development [28].

### Genome structure and encoded proteins

**Figure 2:**
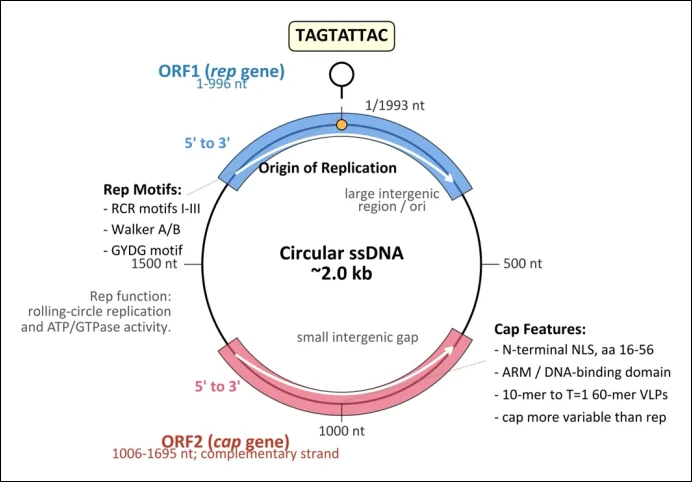
Schematic representation of Beak and feather disease virus (BFDV) genome organization. The approximately 2.0 kb circular ssDNA genome of BFDV is shown with nucleotide scale markers.

BFDV possesses a compact, covalently closed circular ssDNA genome of approximately 2.0 kb arranged in an ambisense orientation. Open reading frame 1 (ORF1), located on the virion-sense strand, encodes the replication-associated protein (Rep), whereas ORF2, located on the complementary strand, encodes Cap [4] (Figure 2). The short intergenic control region contains the origin of replication (*ori*), comprising a stem-loop structure harboring the conserved nonanucleotide motif 5′-TAGTATTAC-3′ [31]. Rep recognizes this origin and introduces a site-specific nick to initiate rolling-circle replication [32]. Rep also possesses experimentally confirmed endonuclease, adenosine triphosphatase (ATPase), and Guanosine triphosphatase (GTPase) activities, which collectively constitute the core enzymatic machinery required for viral genome replication [32, 33].

Cap functions not only as the structural shell of the virion but also as a multifunctional protein involved in nuclear trafficking and genome packaging. Its arginine-rich N-terminal region possesses both nuclear localization and DNA-binding functions that facilitate genome packaging and nuclear transport. Experimental evidence further indicates that Cap enhances Rep activity and facilitates the transport of Rep into the nucleus [31, 33, 34]. Thus, within its limited coding capacity, the BFDV genome accommodates three essential biological functions: replication initiation, capsid-mediated nuclear access, and genome packaging. Subsequent sections discuss how genetic variation and recombination within these functional regions influence diagnostic assay performance and vaccine antigen selection.

## REP PROTEIN STRUCTURE AND FUNCTION

**The BFDV Rep protein consists of two principal functional modules:** an N-terminal origin-cleavage endonuclease module and a C-terminal P-loop/SF3-like nucleoside triphosphatase (NTPase) module. Chen *et al.* [32] experimentally characterized the endonuclease activity responsible for origin nicking. Huang *et al.* [33] subsequently demonstrated that the Walker A and Walker B motifs together with the intervening Glycine–tyrosine–aspartic acid–glycine motif (GYDG) regulate the ATPase and GTPase activities of Rep. Accordingly, the GYDG motif should be considered an NTPase-associated functional determinant, although its structural contribution to full-length Rep assembly and Rep–Cap interactions remains unresolved. Structural interpretation of BFDV Rep therefore continues to rely on conserved functional motifs, biochemical characterization, and comparative structural analyses of circoviruses [28].

The high degree of functional conservation also makes the *rep* gene an attractive molecular diagnostic target. Compared with the highly variable *cap* gene, *rep*-based PCR and high-resolution melting (HRM) assays may improve the detection and genotyping of genetically divergent BFDV strains when appropriate primer design is employed [36].

### Cap protein structure and immunological properties

Cap integrates structural, intracellular trafficking, and antigenic functions within a single multifunctional protein. Comparative structural analyses place BFDV Cap within the conserved jelly-roll β-barrel scaffold required for T = 1 capsid assembly [28]. Experimental evidence demonstrates that Cap binds viral DNA, facilitates the nuclear import of Rep, and utilizes its N-terminal arginine-rich NLS during nuclear trafficking and VLP assembly [31, 34]. High-resolution structural studies further demonstrate that the ARM/NLS region undergoes conformational changes between different assembly states, thereby linking genome binding, nuclear import, and capsid maturation [30]. These structural constraints require Cap to preserve essential assembly and trafficking interfaces while allowing variation within exposed surface loops that interact with the host immune system.

These functional constraints position Cap at the center of both viral evolution and vaccine development. Unlike Rep, which is highly conserved because of its essential roles in origin-cleavage and NTPase activity, Cap exhibits substantially greater sequence diversity among geographically distinct lineages, host-associated genotypes, and even viral populations within individual birds [15, 35, 37]. Such diversity may contribute to antigenic drift and potential vaccine mismatch. However, there is currently no direct evidence demonstrating that specific Cap variants mediate immune escape, neutralization failure, or vaccine breakthrough. VLPs composed of self-assembled Cap multimers closely mimic the native viral capsid, and both plant-based expression systems and recently developed Cap/VLP platforms have demonstrated encouraging immunogenicity together with potential thermostable formulations [18–20, 38]. Nevertheless, these vaccine candidates have yet to demonstrate broad heterologous protection or universal protective efficacy against genetically diverse BFDV strains.

### Potential additional ORFs

Current evidence supporting additional protein-coding capacity within the BFDV genome beyond Rep and Cap remains limited. This differs from PCV type 2 (PCV2), which encodes an accessory ORF3 protein with pro-apoptotic activity [39]. No equivalent accessory gene has been identified in BFDV, and the compact T = 1 capsid architecture imposes strong constraints on genome expansion [1, 32]. Consequently, any additional biological functions are more likely to arise from compact regulatory elements, host-factor interactions, or incompletely characterized short transcripts than from a conserved third major ORF.

The intergenic region is compact but functionally complex, containing overlapping promoter elements together with the viral origin of replication rather than serving as a non-functional spacer sequence [28]. Previous comparative genomic studies documented considerable genetic diversity and Cap variability but failed to identify a conserved third major protein-coding ORF [40]. Consequently, evidence supporting additional coding sequences or regulatory elements remains limited. Future investigations should integrate long-read transcriptomics, direct RNA sequencing, ribosome profiling, quantitative proteomics, and mutational reporter assays to distinguish authentic expressed microproteins and regulatory RNAs from annotation artifacts. Because prediction of small ORFs remains technically challenging and prone to annotation errors [41], comprehensive functional genomic studies should be regarded as an important future research priority rather than an established component of current BFDV biology [42].

### Molecular criteria for the framework

The structural and genomic characteristics of BFDV establish the first two molecular decision layers within the proposed BFDV Resilience Framework. Conserved *rep* motifs provide robust targets for broad molecular screening because of their relatively low sequence variability. In contrast, the greater genetic diversity of *cap*, together with recombination-prone genomic regions, necessitates confirmatory sequencing and antigen-matching analyses when interpreting molecular surveillance results or developing vaccine candidates [16, 30, 36]. Accordingly, routine molecular surveillance should preferentially target conserved genomic regions to maximize diagnostic sensitivity, whereas outbreak investigations and vaccine development programs should incorporate sequencing of *cap*, *rep*, the *ori* stem-loop region, and recombinant breakpoints whenever feasible. Such an integrated molecular approach will improve the identification of emerging variants, strengthen genomic surveillance, and facilitate the rational design of future diagnostic assays and broadly protective vaccine strategies.

## PATHOGENESIS AND CLINICAL MANIFESTATIONS

### Clinical forms of PBFD

The clinical presentation of PBFD varies according to host species, age, immune status, viral burden, and the stage of infection. For clinical interpretation, PBFD is most appropriately classified into four major forms: peracute, acute, chronic, and subclinical [43, 44] (Table 1). Rather than representing a fixed sequence of disease progression, these forms distinguish fulminant juvenile disease from chronic feather and beak disorders, immunosuppressive syndromes, and persistent carrier states.

**Table 1:** Clinical forms of Psittacine beak and feather disease (PBFD), risk stratification, and host susceptibility.

**Category**	**Form/context**	**Key signal**	**Interpretation**	**Risk/outcome**	**Representative host/population**
Clinical	Peracute	Leukopenia/pancytopenia; hepatic necrosis	Systemic disease requiring blood and tissue testing	Rapidly fatal	Juvenile African gray parrots (*Psittacus erithacus*) [45, 46, 54]
Clinical	Acute	Immunosuppression; lymphoid depletion	Age- and species-dependent disease	Guarded to poor prognosis	Young psittacines, particularly juvenile African gray parrots [45, 46, 53]
Clinical	Chronic	Symmetrical feather and beak abnormalities	Detection primarily from feather and skin samples	Progressive clinical deterioration	Cockatoos and other chronically affected psittacine species [49, 55]
Clinical	Subclinical	Asymptomatic persistence or intermittent viral shedding	Repeated multi-matrix testing required	Long-term transmission risk	Wild Australian parrot populations, particularly crimson rosellas [7, 21]
Contextual	Genetic bottleneck	Limited demographic and immunogenetic buffering	Increased spillover and viral evolutionary risk	High conservation risk	Orange-bellied parrots (*Neophema chrysogaster*) [9, 15, 56]
Contextual	Population recovery	Post-detection surveillance	Longitudinal PCR monitoring	Dynamic recovery trajectory	Cape parrots (*Poicephalus robustus*) [10, 57]

The peracute form is most frequently reported in juvenile African gray parrots (*Psittacus erithacus*), in which severe systemic disease progresses rapidly [45, 46]. Clinical manifestations include profound leukopenia, pancytopenia, and hepatic necrosis [45]. This fulminant presentation is consistent with the marked tropism of BFDV for rapidly proliferating lymphoid and hematopoietic tissues, although the precise sequence of events leading to early immune collapse remains incompletely understood.

The acute form occurs predominantly in juvenile and subadult birds. Although BFDV infects a wide range of psittacine species, disease severity and age-related susceptibility differ among hosts [44, 47]. African gray parrots may develop rapid-onset lymphopenia and severe heteropenia, resulting in marked impairment of innate and adaptive immune defenses [45]. As immunosuppression progresses, affected birds become increasingly susceptible to opportunistic pathogens. Cryptosporidiosis in PBFD-affected cockatoos represents one of the best-documented examples of secondary opportunistic infection associated with acute disease [48].

The chronic form represents the classical manifestation of PBFD and is generally recognized over successive molt cycles rather than after the appearance of a single abnormal feather. Progressive, bilaterally symmetrical feather dystrophy, retained feather sheaths, feather shaft constriction or hemorrhage, and premature feather loss reflect persistent viral infection of actively growing feather follicles. In advanced disease, beak overgrowth, cracking, necrosis, or surface deformities may interfere with normal feeding behavior [49, 50]. Histopathological examination supports a primary follicular epithelial disease process characterized by intranuclear and intracytoplasmic viral inclusion bodies within epithelial cells and macrophages, accompanied by apoptosis of infected skin and feather tissues [51, 52]. Chronic infections may persist for months or years; however, prognosis depends less on feather abnormalities alone than on the degree of immunosuppression, husbandry-related stress, and the occurrence of secondary infections [44, 53].

The subclinical form presents the greatest challenge for disease surveillance because clinically normal birds may remain tissue-positive or shed virus intermittently despite the absence of feather or beak abnormalities [21, 48]. Consequently, visual examination and a single negative blood test are insufficient to confirm freedom from infection. Diagnostic classification should instead rely on repeated multi-matrix testing, supported by environmental sampling where available, together with reassessment during periods associated with increased viral shedding, such as molt, transport, breeding, or relocation between facilities. At present, longitudinal predictors that distinguish birds remaining subclinically infected from those progressing to overt clinical disease remain poorly defined.

Species-specific variation in PBFD appears to reflect differences in conservation status, population genetics, and environmental exposure rather than representing fixed clinical categories. Orange-bellied parrots (*Neophema chrysogaster*) provide one of the clearest examples among endangered psittacine species. BFDV has been detected in PBFD-affected wild fledglings from the remnant critically endangered population, with evidence supporting genetically distinct viral lineages and spillover from sympatric parrot species [9]. Subsequent whole-genome investigations demonstrated repeated viral introductions into this immunologically vulnerable population [56], while longitudinal genomic analyses revealed rapid viral evolution characterized by frequent mutation, recombination, and the emergence of multiple intra-host variants [15].

Cape parrots (*Poicephalus robustus*) present a contrasting epidemiological scenario. Comparative genomic analyses of viruses recovered from captive and wild birds suggested independent infection events and potential transmission across the captive–wild interface [57]. Nevertheless, subsequent longitudinal field investigations documented evidence of population-level recovery following BFDV infection [10]. Collectively, these findings demonstrate that host species, conservation status, demographic history, and population structure should be incorporated into clinical risk assessment rather than relying solely on clinical presentation to predict disease outcome.

Cape parrots (*Poicephalus robustus*) present a contrasting epidemiological scenario. Comparative genomic analyses of viruses recovered from captive and wild birds suggested independent infection events and potential transmission across the captive–wild interface [57]. Nevertheless, subsequent longitudinal field investigations documented evidence of population-level recovery following BFDV infection [10]. Collectively, these findings demonstrate that host species, conservation status, demographic history, and population structure should be incorporated into clinical risk assessment rather than relying solely on clinical presentation to predict disease outcome.

### Immunosuppression and secondary infections

Immunosuppression is a hallmark of BFDV infection and is believed to result primarily from viral injury to rapidly proliferating epithelial, hematopoietic, and lymphoid tissues rather than from a single, well-defined immune-evasion mechanism [53]. Because BFDV encodes the replication-associated protein (Rep) but lacks an endogenous DNA polymerase, viral genome replication depends entirely on the host cellular DNA replication machinery. Consequently, tissues with a high proportion of cells in the S phase of the cell cycle, including feather follicles, bone marrow, thymus, and the bursa of Fabricius, represent biologically plausible sites of viral replication [32, 53]. In severe juvenile infections, this tissue tropism manifests clinically as leukopenia or pancytopenia and pathologically as hepatic necrosis and lymphoid tissue injury, particularly in African gray parrots (*Psittacus erithacus*) [45]. Histopathological studies have additionally reported extracutaneous viral inclusion bodies, bursal atrophy, lymphocytic depletion, multifocal tissue necrosis, and macrophage infiltration [46, 58].

From a mechanistic perspective, BFDV-associated immunosuppression is more accurately characterized as a combination of lymphoid destruction, tissue compartmentalization, and host genetic influences rather than a single virus-mediated interferon antagonism pathway. Viral replication within lymphoid tissues, feather follicles, and the skin disrupts the cellular architecture required for B-cell maturation, T-cell activation, antigen presentation, and macrophage-mediated clearance of pathogens [7, 53]. Moreover, viral replication may persist within feather follicles and cutaneous tissues even after blood viremia has declined, creating an anatomical reservoir in which circulating antibodies may not accurately reflect local viral activity [7, 59]. Population-based studies further suggest that host genetic factors influence infection outcome. For example, red-crowned parakeets exhibited evidence of positive selection at the Toll-like receptor 3 (*TLR3*) locus as BFDV prevalence declined, whereas studies in crimson rosellas demonstrated associations among host heterozygosity, genotype rarity, infection probability, and viral load [26, 60]. Nevertheless, direct experimental evidence confirming activation of *TLR3*-mediated signaling pathways during BFDV infection remains unavailable.

As adaptive immune function progressively deteriorates, susceptibility to opportunistic infections becomes a major determinant of clinical outcome [53]. Cryptosporidiosis in PBFD-affected cockatoos represents one of the best-documented examples of opportunistic disease associated with severe immunosuppression [48]. At this stage, clinical deterioration extends beyond feather and beak abnormalities and becomes a systemic disease process. However, direct molecular evidence demonstrating BFDV-mediated suppression of interferon-stimulated genes, antigen-processing pathways, or major histocompatibility complex (MHC) class I and class II expression remains limited. Future investigations should combine quantitative viral load measurements with transcriptomic analyses of *TLR3*, interferon-β (IFN-β), MHC-I, MHC-II, cytokine expression, and apoptosis-related pathways using matched samples collected from blood, the bursa of Fabricius, spleen, skin, and feather follicles.

Co-infection represents both a downstream consequence and a potential amplifier of BFDV-induced immunosuppression. Early clinical reports documented cryptosporidiosis in PBFD-affected cockatoos [48]. More recently, multiplex molecular surveillance has demonstrated that exposure to multiple viral pathogens is common in captive and traded psittacine populations. In Italy, a survey of 319 non-traditional companion birds reported BFDV and avian polyomavirus type 1 (APV-1) detection rates of 13.79% and 2.19%, respectively, with five confirmed BFDV/APV-1 co-infections and a significant association between the two viruses (p < 0.001). Psittacid herpesvirus type 1 (PsHV-1) and avian metapneumovirus (aMPV) were not detected in that study population [61]. Similarly, among clinically healthy traded psittacine birds in Thailand, psittacine bornavirus (PaBV), BFDV, and APV were detected in 7.17%, 7.94%, and 0.57% of samples, respectively, whereas viral co-infections occurred in 0.52% of birds. Among virus-positive individuals, co-infection frequencies were highest in APV-positive birds (24.14%), followed by PaBV-positive (8.88%) and BFDV-positive birds (6.81%) [62]. Collectively, these findings demonstrate frequent viral co-circulation and overlapping transmission pathways. Nevertheless, quantitative evidence demonstrating that BFDV directly increases viral replication or disease severity associated with APV, PaBV, PsHV-1, or other herpesviruses remains scarce. Addressing this knowledge gap will require longitudinal qPCR-based investigations integrating viral load measurements with standardized clinical severity scores.

### Viral load and disease progression

Viral load frequently correlates with disease severity; however, tissue compartmentalization substantially complicates clinical interpretation. High viral loads detected in blood or lymphoid tissues are generally indicative of acute systemic infection, particularly in juvenile birds [42]. Conversely, population-based surveillance has shown that apparently healthy wild parrots may shed low concentrations of virus intermittently despite the absence of clinical disease [63]. Diagnostic sensitivity also differs markedly among sample types, with blood performing differently from feather and cloacal swab samples [64]. Furthermore, detection of circulating antibodies should not be interpreted as evidence of viral clearance because humoral immune responses may coexist with persistent viral DNA or declining but detectable viremia [59, 65].

Recent multi-tissue investigations illustrate these diagnostic limitations. In a study of 66 clinically healthy wild crimson rosellas, BFDV DNA was detected in at least one tissue in 94% of individuals [7]. Among subadult birds, blood samples were positive in 74% of cases, whereas positivity rates across most non-fecal tissues ranged from 76% to 87%. In contrast, none of the adult birds tested positive in blood (0/34), despite persistent viral detection in tissues, particularly the spleen, lung, and skin [7]. Of the 42 birds that tested negative by blood PCR, 38 were positive in at least one tissue. Consequently, reliance on blood samples alone would have produced an apparent prevalence of only 35.3%, with an estimated diagnostic sensitivity of 37%. By comparison, skin samples alone achieved a sensitivity of 72%, whereas the combination of skin and blood feather samples increased sensitivity to 92%. These findings demonstrate that blood, feather, skin, cloacal or fecal swabs, serological testing, and environmental sampling should be regarded as complementary diagnostic approaches rather than interchangeable sample matrixes. This principle is particularly relevant when nest-box swabs or environmental dust are used to assess site-level contamination [8].

Host age and husbandry conditions further influence disease progression. Juveniles infected during the period of bursal development frequently develop severe lymphoid depletion, whereas immunocompetent adults may suppress circulating viremia while maintaining persistent viral infection within tissues. Environmental stressors including overcrowding, nutritional deficiencies, transportation, breeding activity, and exposure to mixed microbial communities may exacerbate clinical disease or increase viral shedding. However, the magnitude of these effects has not yet been quantified and warrants investigation through longitudinal studies [53, 66, 67]. Consequently, prognostic assessment should integrate clinical examination, multi-matrix qPCR, serological findings, environmental surveillance, and repeated sampling over time rather than relying on a single blood test.

Longitudinal investigations also demonstrate that viral shedding is highly dynamic. Approximately 56% of shedding-positive wild parrots excrete virus intermittently rather than continuously [21]. In wild crimson rosellas, BFDV detection rates were significantly higher outside the breeding season than during the breeding season (43.2% versus 10.1%) [22]. Environmental surveillance has produced similar findings. Nest-box eDNA swabs were positive in 36.4% of nest boxes occupied by infected birds and in 80% of nests containing actively shedding parents. Viral DNA remained detectable in nest boxes for up to 3.7 months after occupancy [8]. These observations demonstrate that classification of birds simply as infected or uninfected is biologically inadequate. Instead, decisions regarding quarantine-release, flock management, and facility decontamination should be based on repeated multi-matrix surveillance rather than single-point diagnostic testing.

### Clinical risk translation

Current evidence on BFDV pathogenesis provides a practical framework for clinical risk assessment. Within the proposed BFDV Operational Risk Score, risk should increase in birds exhibiting peracute juvenile disease, high viral loads, multi-matrix positivity, persistent tissue infection despite negative blood PCR results, or documented intermittent viral shedding. Conversely, antibody positivity alone should not be interpreted as evidence of viral clearance because seroconversion may occur despite persistent tissue infection [7, 21, 65]. Accordingly, supportive treatment, isolation procedures, repeated diagnostic sampling, and environmental verification should be implemented as integrated components of both clinical management and biosecurity programs rather than being considered independent interventions.

## EPIDEMIOLOGY AND GLOBAL SPREAD

### Trade-driven dispersal and phylogeographic patterns

**Figure 3:**
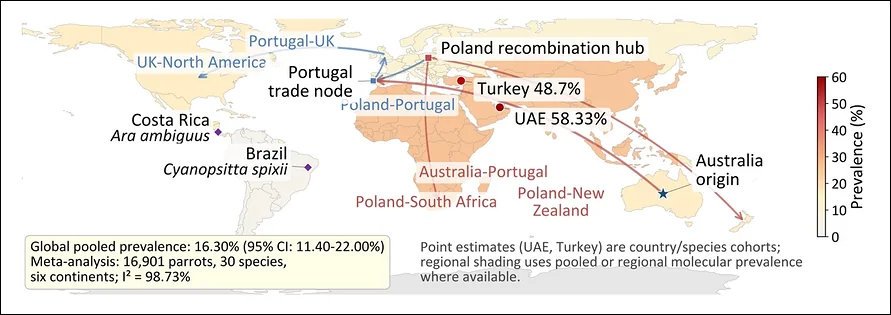
Global molecular prevalence and phylogeographic dispersal of BFDV. Regional shading indicates pooled molecular prevalence estimates, whereas point annotations identify representative country- or species-specific cohorts (e.g., United Arab Emirates, 58.33%; Türkiye, 48.7%) [75, 76]. The global baseline prevalence (16.30%; 95% CI: 11.40%–22.00%; I² = 98.73%) is derived from the 2025 meta-analysis by Zhang *et al.* [71]. Arrows indicate Bayesian-supported viral migration pathways connecting major commercial trade routes and recombination hotspots [68, 69]. Diamond symbols denote conservation sentinel and reintroduction monitoring sites for the great green macaw (*Ara ambiguus*) and Spix's macaw (*Cyanopsitta* *spixii*) [11, 12].

PBFD was first formally recognized in Australian psittacine birds during the 1970s. Nevertheless, earlier reports describing feather abnormalities in declining *Psephotus* populations suggest that a clinically similar disease may have existed before its formal description [44]. Phylogeographic analyses identify Australia as an important historical source region for BFDV. However, the contemporary global distribution of the virus is more consistently explained by repeated legal and illegal movements of parrots than by natural dispersal alone [68]. International trade in psittacine birds has therefore become the principal driver of global BFDV dissemination, particularly when birds are transported without appropriate BFDV screening or quarantine measures [6] (Figure 3).

Bayesian phylogeographic analyses of 184 complete BFDV genomes demonstrate that viral dissemination is considerably more complex than a simple outward expansion from native psittacine ranges. Several inferred transmission routes indicate viral movement from Europe back into regions where psittacine species are indigenous, illustrating bidirectional dissemination associated with international bird trade [68]. Captive breeding programs may further accelerate viral evolution by maintaining genetically divergent viral lineages in close proximity. For example, molecular characterization of 43 infected birds from 18 breeding facilities in Poland demonstrated extensive viral recombination and high genetic diversity, indicating that captive populations may function as important reservoirs for viral evolution [69]. Similar evidence has been reported from invasive parakeet populations in Spain, where BFDV genotypes clustered more closely with strains originating from geographically distant regions than with local viral lineages, strongly supporting trade-mediated introduction rather than natural long-distance dispersal [70].

A recent global meta-analysis provides the most comprehensive quantitative assessment of BFDV distribution to date. Zhang *et al.* [71] analyzed data from 30 molecular epidemiological studies published between 2003 and 2024, encompassing 16,901 parrots representing 30 species from 34 countries. The pooled global molecular prevalence of BFDV was estimated at 16.30% (95% confidence interval [CI]: 11.40%–22.00%) [71]. To further explore epidemiological trends, we conducted an exploratory aggregate-data reanalysis of published subgroup frequencies from this meta-analysis. Crude odds ratio (OR) estimates indicated significantly higher BFDV detection in juvenile than adult birds (OR = 2.81; 95% CI: 2.05–3.85), in cloacal swabs compared with blood samples (OR = 3.31; 95% CI: 2.62–4.17), during spring compared with summer (OR = 5.89; 95% CI: 4.28–8.12), and in *Agapornis* species compared with *Ara* species (OR = 4.46; 95% CI: 3.03–6.57). Similarly, analysis of the Thailand trade cohort demonstrated substantially higher crude odds of BFDV detection in the family Psittaculidae than in Psittacidae (OR = 7.46; 95% CI: 6.19–9.00), suggesting a strong influence of host family and trade-associated factors [62]. Independent evidence from Italy further identified public exhibition as a significant risk factor for BFDV detection (OR = 2.93; 95% CI: 1.25–6.48; p = 0.009) [61]. These estimates should be interpreted as hypothesis-generating rather than definitive because important confounding factors, including study design, geographic region, diagnostic sample type, host age, captive or trade status, and seasonal or climatic influences, were not controlled. Likewise, robust estimates of clade-specific pathogenicity or climate-associated transmission cannot yet be generated because paired full-genome, phenotypic, and environmental datasets remain limited. Nevertheless, these findings strongly support risk-based surveillance strategies focused on commercial trade hubs, bird exhibitions, and mixed-species aviaries.

Unregulated and informal wildlife markets represent an additional biosecurity concern because information regarding bird origin, quarantine history, health certification, and interspecific contact is frequently unavailable or incomplete. Analysis of Indonesian Facebook-based wildlife trading documented 283 advertisements involving 861 parrots distributed across 38 online trading groups, although many transactions subsequently moved to private communication platforms such as WhatsApp, limiting traceability [72]. These observations emphasize the need for targeted surveillance of online wildlife trade networks. However, genotype-confirmed transmission studies are still required to directly link specific BFDV introductions with individual online trade pathways.

### Conservation and stress interaction

Recent conservation experiences demonstrate that even isolated detections of BFDV may threaten endangered species recovery programs, despite uncertainty regarding transmission pathways. Two free-ranging great green macaws (*Ara ambiguus*) in Costa Rica were diagnosed with PBFD, raising concern because the global wild population is estimated to comprise only 500–1,000 mature individuals [11]. In populations of this size, even sporadic viral detection warrants comprehensive landscape-level surveillance encompassing nest cavities, naturally shed feathers, surrounding captive collections, and sympatric psittacine populations before translocation or reinforcement decisions are implemented. eDNA surveillance of nest boxes offers a practical and non-invasive approach for supporting these conservation activities [8].

Comparable biosecurity challenges exist for the reintroduction program of the Spix's macaw (*Cyanopsitta* *spixii*) within Brazil's Caatinga biome. Population viability modeling, annual supplementation, habitat restoration, and long-term coexistence planning already constitute integral components of this recovery program [12, 73]. Although no peer-reviewed evidence of a BFDV outbreak has been reported in the reintroduced population, its limited founder population and reliance on repeated releases make comprehensive pathogen surveillance an essential component of conservation planning. Accordingly, BFDV screening should be performed before release, repeated during post-release monitoring, and integrated with environmental sampling of nest sites and release habitats.

Ecological stressors may further modify BFDV transmission dynamics without necessarily demonstrating direct climatic causation. Seasonal variation in BFDV prevalence is likely influenced by a combination of breeding activity, molting, host aggregation, sampling design, and climatic variables rather than by any single environmental factor [71]. In small, intensively managed populations, disease impacts may also interact with supplementary feeding programs and other conservation interventions in unexpected ways [74]. Future investigations should therefore evaluate drought, heat stress, food limitation, artificial feeding, and nest-site scarcity as potential modifiers of viral exposure and transmission. Such relationships should be investigated using integrated longitudinal datasets combining BFDV qPCR, environmental DNA surveillance, demographic monitoring, and movement ecology rather than inferred solely from ecological associations.

### Environmental persistence and transmission dynamics

The remarkable environmental stability of BFDV makes effective control of fomite-mediated transmission a cornerstone of disease prevention. Circoviruses are generally resistant to physical and chemical inactivation [39], and early hemagglutination (HA) studies demonstrated that BFDV remained detectable after exposure to 80°C for 30 min [77]. Environmental surveillance further indicates that viral DNA can persist within occupied nest boxes even after birds have vacated the nesting site [8]. Consequently, nest-box swabs and feather dust samples represent practical, non-invasive tools for environmental surveillance, particularly in endangered or stress-sensitive populations. Nevertheless, detection of viral DNA in environmental samples indicates environmental contamination or previous exposure rather than active infection in a particular bird.

**Figure 4:**
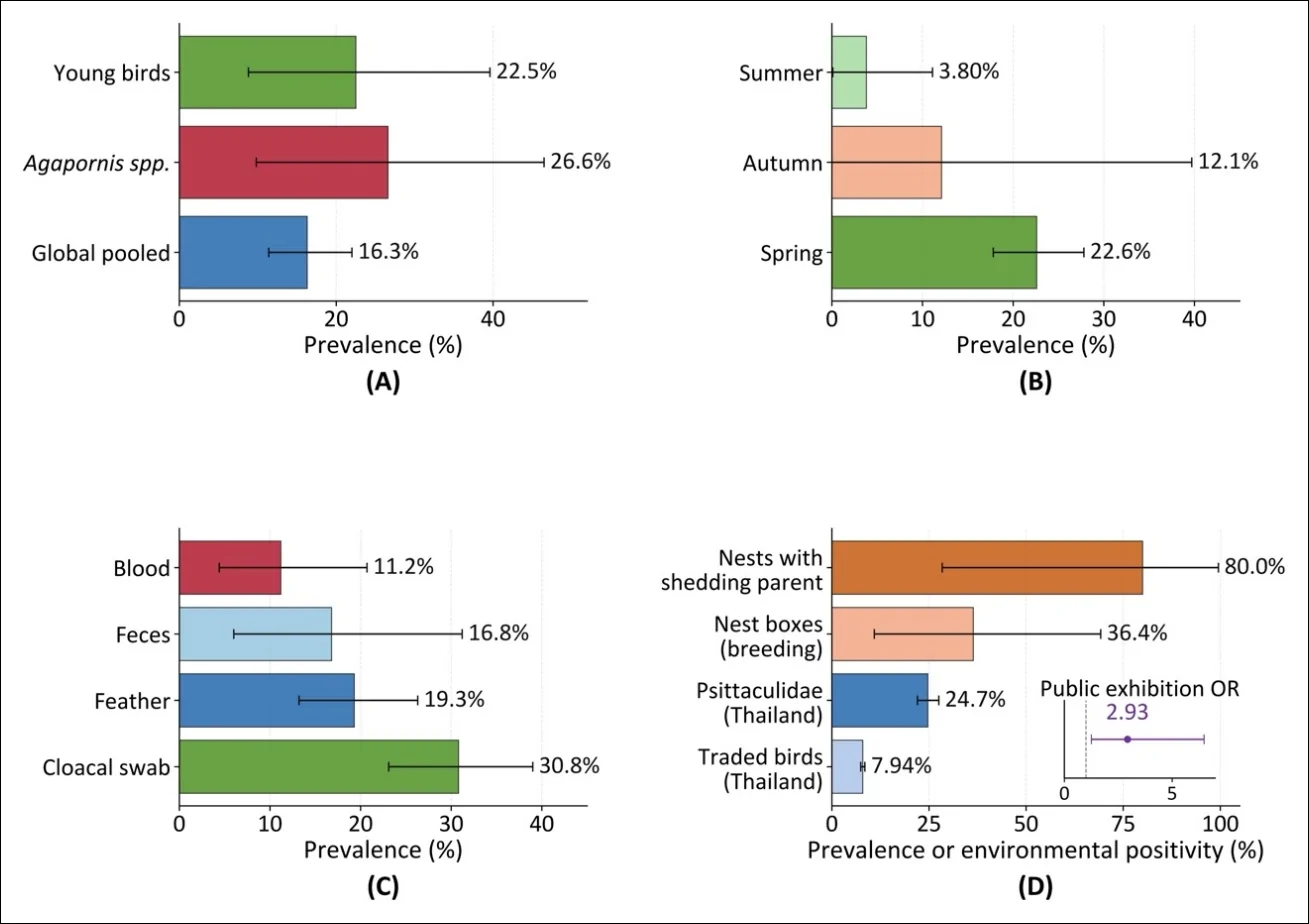
Subgroup molecular prevalence and trade- and environmental-associated risk metrics for Beak and feather disease virus (BFDV). (A) Molecular prevalence stratified by host genus and age class relative to the global pooled prevalence [71]. (B) Seasonal variation in BFDV prevalence among spring, autumn, and summer cohorts [71]. (C) Comparative diagnostic sensitivity of blood, fecal, feather, and cloacal swab sample matrixes [71]. (D) Operational surveillance metrics incorporating live-bird market monitoring [62], nest-box environmental DNA (eDNA) surveillance [8], and a forest plot inset illustrating the multivariable odds ratio for public exhibition as a risk factor for BFDV detection (OR = 2.93; 95% CI: 1.25–6.48; p = 0.009) [61]. Error bars represent 95% CI.

Accordingly, BFDV transmission is primarily driven by exposure to contaminated feather dust, feces, crop secretions, nest materials, husbandry equipment, and other fomites [2, 78]. Detection rates are generally highest during spring and autumn and lowest during summer. However, these seasonal trends are more likely to reflect the combined influences of breeding activity, molting, host aggregation, sampling strategies, and climatic conditions than the direct effects of temperature alone [71]. BFDV DNA has also been identified in embryonated eggs, suggesting that vertical transmission may occur under some circumstances [79]. Despite this possibility, current evidence indicates that horizontal transmission through contaminated environments remains the principal target for biosecurity interventions in breeding facilities, rehabilitation centers, and natural nesting habitats [8, 78]. Ectoparasites have additionally been proposed as potential mechanical carriers of BFDV, although convincing evidence demonstrating biological vector competence or active virus transmission is currently lacking [80] (Figure 4).

### Host range expansion and cross-species transmission

BFDV is primarily a pathogen of birds belonging to the order Psittaciformes. However, molecular detection of viral DNA in several non-psittacine avian orders has raised important questions regarding viral exposure, spillover events, and the potential for host switching [43]. Among currently available evidence, the strongest indication of a genuine host-switch event comes from the rainbow bee-eater (*Merops ornatus*), in which molecular analyses suggest successful cross-order transmission of BFDV [24]. In contrast, BFDV DNA has also been detected in predatory birds, including the red goshawk (*Erythrotriorchis radiatus*) and the powerful owl (*Ninox strenua*). In the absence of evidence demonstrating viral replication within tissues or subsequent viral shedding, these findings are more plausibly explained by trophic exposure following ingestion of infected psittacine prey rather than productive infection [13, 81].

Broader molecular surveys of Australian non-psittacine birds have expanded the known range of species exposed to BFDV but do not establish the existence of multiple independent reservoir hosts [14]. Although spillover events and circovirus genetic exchange may generate viral variants subject to host-specific selection, definitive evidence of host adaptation requires demonstration that the recipient species supports productive viral replication, tissue tropism, viral shedding, and sustained transmission among conspecifics [82] (Table 2). The mechanisms underlying spillover remain poorly resolved because most studies cannot distinguish among trophic acquisition of viral DNA, environmental contamination, mechanical carriage, limited abortive replication, and fully host-adapted infection. Future investigations should therefore integrate rigorous environmental controls with tissue-specific qPCR, assays targeting replicative viral intermediates, *in situ* hybridization or immunohisto-chemistry, serological analyses, longitudinal sampling, and whole-genome sequencing to distinguish transient exposure from productive infection and to clarify the biological mechanisms underlying cross-species transmission.

**Table 2:** Evidence tiers for Beak and feather disease virus detection in non-psittacine avian species.

**Species**	**Order/family**	**Geographic location**	**Clinical signs observed**	**Probable transmission route**
*Corvus coronoides* (Australian raven)	Passeriformes/Corvidae	Australia	Yes (feather disease) [14]	Environmental or fomite-mediated exposure
*Zosterops simplex* (Swinhoe's white-eye)	Passeriformes/Zosteropidae	Hong Kong	No (viral DNA detection only) [66]	Environmental or fomite-mediated exposure associated with captive contact
*Erythrotriorchis radiatus* (Red goshawk)	Accipitriformes/Accipitridae	Australia	No (viral DNA detection only) [13]	Trophic exposure through ingestion of infected prey (potential gastrointestinal pass-through)
*Merops ornatus* (Rainbow bee-eater)	Coraciiformes/Meropidae	Australia	No (viral DNA detection only) [24]	Environmental or fomite-mediated exposure, or trophic exposure through prey ingestion
*Columba livia domestica* (Domestic pigeon)	Columbiformes/Columbidae	Ahvaz, Iran	No (viral DNA detection only) [83]	Environmental or fomite-mediated exposure through contaminated psittacine droppings

Across these reports, detections of BFDV in non-psittacine birds are concentrated primarily in Australia and parts of Asia. This distribution most likely reflects intensive surveillance efforts in Australasia and regional sampling intensity rather than genuine ecological limits to viral distribution. Current evidence indicates that environmental exposure, trophic acquisition, transient infection, and occasional spillover associated with pathological lesions can occur in non-psittacine species. However, there is currently no convincing evidence supporting the existence of multiple established non-psittacine reservoir hosts for BFDV [14, 84].

Demonstration of productive infection requires substantially stronger evidence than the detection of viral DNA alone. Such evidence should include increasing viral loads within biologically relevant target tissues, compatible histopathological lesions, localization of viral nucleic acids or antigens by *in situ* hybridization or immunohistochemistry, seroconversion indicative of an active immune response, repeated detection in the same individuals over time, and phylogenetic evidence supporting sustained transmission within the recipient host species. Collectively, these criteria provide a robust framework for distinguishing incidental viral exposure from genuine host adaptation and productive cross-species transmission.

### External-risk translation

The epidemiological evidence summarized in this review provides the foundation for the external-risk component of the proposed BFDV framework. Enhanced surveillance should be prioritized at international trade hubs, online wildlife trade networks, and other pathways associated with legal and illegal bird movements [6, 72]. Additional surveillance efforts should target public bird exhibitions, mixed-species aviaries, breeding facilities, contaminated nesting environments, and wildlife rehabilitation centers where opportunities for viral transmission are increased [8, 61, 62]. Conservation translocation and reintroduction programs involving threatened psittacine species should likewise incorporate comprehensive pre-release, post-release, and environmental monitoring as integral components of disease risk management [11, 73]. Finally, detections of BFDV in non-psittacine birds should be interpreted according to the strength of available evidence and classified within appropriate evidence tiers rather than being considered proof of established reservoir hosts. This evidence-based approach will improve epidemiological interpretation while minimizing unsupported assumptions regarding host adaptation and cross-species maintenance of BFDV.

## MOLECULAR EVOLUTION AND GENOTYPIC DIVERSITY

### Phylogenetic classification

BFDV exhibits substantial genetic diversity, particularly within the capsid protein -encoding *cap* gene, where amino acid variation is sufficient to influence phylogenetic classification, hypotheses regarding host adaptation, and potential antigenic matching [82]. The most comprehensive classification to date analyzed 454 complete BFDV genomes and resolved the virus into two major phylogenetic clades, Genotype I (GI) and Genotype II (GII). Clade GI was further divided into six subclades, whereas GII comprised two subclades. Analysis of the same dataset also identified 27 recombination events distributed across both the *rep* and *cap* coding regions [16]. BFDV evolves at an estimated rate of 3.41 × 10⁻³ substitutions/site/year, exceeding the commonly reported evolutionary rates for PCV2 and PCV type 3 [15, 85, 86]. Recombination is likewise non-random, with complete-genome analyses and breeding-facility isolates demonstrating clustering of recombination breakpoints near the origin of replication, the *rep–cap* junction, and hypervariable regions within the *cap* gene [16, 69].

The biological consequences of this genetic diversity remain largely inferential because most variants have not been evaluated experimentally. Amino acid substitutions within exposed Cap surface loops may influence antibody recognition, receptor interactions, or host specificity, whereas the internal structural scaffold of the capsid appears to be under stronger evolutionary constraint [30, 82]. Conversely, mutations that compromise Rep function, the origin of replication, capsid assembly, nuclear localization, or genome packaging are expected to be removed through purifying selection. This evolutionary asymmetry provides a plausible explanation for why antigenic drift is more likely to arise through variation or recombination within the Cap protein than through alterations affecting essential replication functions. Nevertheless, the vaccine relevance of individual variants cannot be inferred solely from sequence divergence and requires confirmation through experimental challenge studies or virus neutralization assays [18, 19].

Phylodynamic analyses further demonstrate that viral diversification is closely associated with international bird trade. Bayesian reconstruction based on 184 complete BFDV genomes identified six statistically supported migration pathways linking Poland, Portugal, the United Kingdom, North America, Japan, and China, together with additional connections involving South Africa, Australia, and New Zealand [68]. Poland and Portugal emerged as highly connected transmission hubs, consistent with sustained viral movement through captive breeding and commercial trade networks. These inferred pathways represent statistically supported viral migration events rather than direct measures of bird trade volume. Consequently, phylogeographic analyses should be interpreted alongside trade permits, wildlife seizure records, and breeding-facility surveillance data to accurately reconstruct transmission pathways.

### Regional genotype divergence and clinical implications

**Figure 5:**
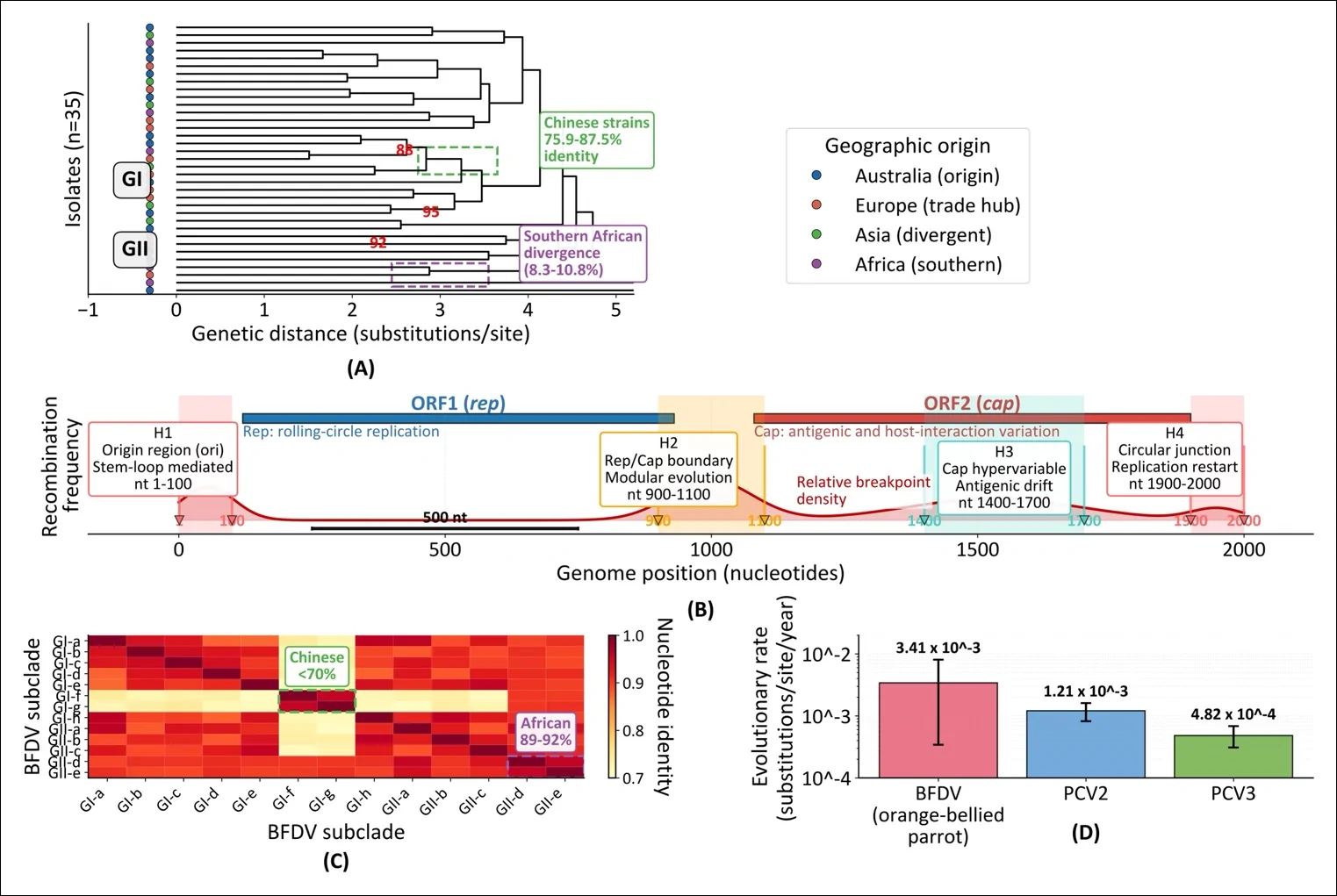
Molecular evolution and recombination landscape of BFDV. (A) Geographic distribution of phylogenetic clades GI and GII, highlighting divergent Chinese isolates (75.9%–87.5% nucleotide identity; green) and southern African strains (8.3%–10.8% divergence; purple) [16, 87]. (B) Circular BFDV genome (~2.0 kb) illustrating four major recombination hotspots: H1 (1–100 nt; origin of replication), H2 (900–1100 nt; *Rep-Cap* junction), H3 (1400–1700 nt; hypervariable *cap* region), and H4 (1900–2000 nt; circular genome junction). Functional genomic regions include ORF1 (*rep*; 120–930 nt; blue) and ORF2 (*cap*; 1080–1900 nt; red) [16, 27, 69]. (C) Heatmap showing nucleotide identity across 13 phylogenetic subclades (70%–100%), emphasizing divergence among Chinese (GI-f and GI-g) and southern African (GII-d and GII-e) lineages. (D) Estimated evolutionary rates expressed as substitutions per site per year.

The extensive genetic diversity of BFDV presents significant challenges for both molecular diagnosis and vaccine development. Chinese isolates share only 75.9%–87.5% nucleotide identity with established reference strains and cluster within distinct phylogenetic lineages, raising concern that PCR assays designed against conserved reference genomes may fail to detect genetically divergent variants [16, 87]. Vaccine development faces similar obstacles because the Cap protein exhibits substantial sequence variability among circulating strains [82]. Consequently, immune responses elicited by one viral lineage may provide incomplete protection against genetically divergent variants from other geographical regions. However, this possibility remains hypothetical because robust experimental evidence demonstrating reduced cross-protection is currently unavailable. Comprehensive molecular surveillance combined with cross-clade immunological evaluation is therefore required to determine whether future vaccines should target region-specific lineages or broadly conserved antigens (Figure 5).

From a vaccine development perspective, the principal challenge has shifted beyond sequence diversity to antigenic diversity. Comprehensive BFDV antigenic cartography and cross-neutralization matrices spanning both GI and GII clades are not yet available, largely because routine BFDV culture systems and standardized virus neutralization assays remain lacking. Historical hemagglutination (HA) and hemagglutination inhibition (HI) assays, together with more recent VLP-based indirect enzyme-linked immunosorbent assay (iELISA) platforms, demonstrate broad serological reactivity among BFDV strains. However, these assays evaluate antibody binding or hemagglutination inhibition rather than protective virus neutralization across genetically divergent lineages [63, 77]. Consequently, development of multivalent VLP-based vaccines represents a scientifically plausible strategy but should currently be regarded as a hypothesis rather than an evidence-based requirement. Experimental data comparing homologous and heterologous challenge following vaccination remain unavailable for contemporary vaccine platforms [18–20]. To date, evidence of protective immunity is largely restricted to early challenge studies using inactivated whole-virus vaccines [25].

Interpretation of genotype-specific virulence also requires considerable caution. Although severe or atypical disease has been reported in particular clinical settings, including peracute infections in African gray parrots, existing studies rarely control for important confounding variables such as host species, age, immune status, tissue distribution of virus, concurrent infections, and husbandry conditions [45, 54]. Current evidence therefore supports the conclusion that viral genotype may influence antigenicity, tissue tropism, diagnostic performance, and host range. However, convincing evidence demonstrating that any individual phylogenetic clade consistently exhibits greater virulence than others is presently lacking. Future investigations should integrate complete viral genome sequencing with quantitative viral load measurements, tissue tropism analyses, immune profiling, co-infection assessment, and standardized pathological lesion scoring to establish robust genotype–phenotype relationships and accurately define the contribution of viral genotype to disease severity.

### Conceptual models for BFDV ecology and evolution

The evidence synthesized in this review supports the development of three conceptual models that extend beyond descriptive summaries to generate testable hypotheses regarding BFDV ecology and evolution. These frameworks are not intended to represent established biological mechanisms but rather to integrate current knowledge of host immunogenetics, viral recombination, trade-associated phylogeography, and cross-order spillover into practical questions for future surveillance and experimental research.

**Model 1: Host-virus co-evolutionary arms race and balanced polymorphism:** The host-virus co-evolutionary model proposes that BFDV exists within two contrasting epidemiological states. In long-established wild host populations, host immune diversity and viral genetic diversification may achieve a dynamic equilibrium in which persistent infection is maintained without causing sustained population decline. In contrast, naïve, genetically bottlenecked, or trade-stressed populations may experience rapid viral amplification, prolonged subclinical shedding, and increased spillover risk despite infection with genetically similar viruses. This hypothesis is supported by three independent observations. First, red-crowned parakeets exhibited a progressive decline in BFDV prevalence over five years accompanied by evidence of positive selection at the *TLR3* locus [60]. Second, hybrid crimson rosellas exhibited lower BFDV prevalence and reduced viral loads than either parental subspecies [88]. Third, clinically healthy crimson rosellas maintained extensive tissue reservoirs despite frequent negative blood PCR results [7]. Collectively, these observations suggest that host genetic diversity may influence viral persistence and transmission. The model predicts that longitudinal studies integrating *TLR* and MHC genotyping with repeated tissue sampling and viral shedding measurements will identify host genetic markers associated with persistence, viral load, and transmission potential.

**Model 2: Captive-facility mixing-vessel hypothesis:** The captive-facility mixing-vessel hypothesis proposes that breeding facilities and commercial trade networks function as evolutionary compartments in which viral diversification is accelerated. High host density, multispecies housing, repeated introduction of birds from different geographical origins, and chronic physiological stress collectively increase opportunities for co-infection, recombination, and dissemination of genetically novel viral variants. This hypothesis is supported by three complementary observations: extensive recombination detected among isolates from Polish breeding facilities [69], recurrent recombination identified through global whole-genome analyses [16], and Bayesian phylodynamic evidence linking major international trade hubs [68]. The model predicts that facilities characterized by high bird turnover, multiple source countries, and mixed-species collections will exhibit increased haplotype diversity and a greater frequency of recombinant genomes. Furthermore, it predicts that future vaccine evaluations should extend beyond antibody titers to include analyses of Cap hypervariable loop diversity, breadth of antibody binding, viral shedding, and vaccine breakthrough infections.

**Model 3: Spillover continuum from exposure to reservoir establishment:** The spillover continuum provides a conceptual framework for interpreting BFDV detection in non-psittacine species. It divides spillover into four sequential stages: environmental or trophic exposure, transient infection, dead-end spillover associated with clinical disease, and establishment of a competent reservoir host. This framework transforms the evidence hierarchy summarized in Table 2 into a structured sequence of testable surveillance endpoints. Based on current observations, the model predicts trophic exposure without viral shedding in predatory birds, repeated tissue-associated detections in passerine species, and either transient or partially adapted replication in coraciiform birds [13, 14, 24]. Progression toward reservoir status should require progressively stronger evidence, including demonstration of productive viral replication within target tissues, sustained viral shedding, seroconversion, host-specific phylogenetic clustering, and confirmed transmission among conspecific individuals. Application of this framework will facilitate discrimination between incidental viral exposure and genuine host adaptation.

### Bridging molecular insights to applied control

Current understanding of BFDV molecular evolution has direct implications for disease control and surveillance. Viral genetic diversity creates three principal operational challenges. First, sequence divergence may reduce PCR primer-binding efficiency, potentially compromising molecular diagnosis [36]. Second, substantial variability within the Cap protein may limit the breadth of protection provided by Cap-based vaccine candidates across divergent phylogenetic lineages [16]. Third, mixed-species captive collections provide favorable conditions for co-infection and recombination, promoting continued viral diversification [69]. Accordingly, effective disease control should integrate broad-range PCR or qPCR assays with HRM analysis or sequence confirmation [17, 36], repeated multi-matrix and environmental surveillance [7, 8], evidence-based evaluation of vaccine candidates [18, 19], and biosecurity policies specifically targeting international bird trade and wildlife movement [6, 89].

### Genomic risk translation

Molecular evolution provides the foundation for the genomic risk component of the proposed BFDV framework. Detection of recombinant or highly divergent viral genomes should trigger escalation from routine surveillance to enhanced or high risk response, particularly within major international trade hubs, captive breeding facilities, conservation translocation programs, and populations of threatened psittacine species [16, 68, 69]. Accordingly, viral genome sequencing should be incorporated not only as a retrospective phylogenetic tool but also as a prospective component of quarantine decision-making, vaccine strain selection, molecular surveillance, and CITES-linked risk communication. Integrating genomic information into operational disease management will strengthen early detection of emerging variants and improve evidence-based responses to BFDV at both local and international scales.

## DIAGNOSTIC ADVANCES

### Molecular diagnostic platforms

Molecular diagnostic methods have become the cornerstone of routine BFDV detection, complementing traditional approaches such as histopathology and hemagglutination (HA) assays in both clinical and surveillance settings. Conventional PCR remains valuable as an initial screening method, particularly when primers target conserved regions of the *rep* gene [90]. TaqMan qPCR provides substantially greater analytical sensitivity and, in validated assays, can detect as few as 10–30 viral copies per reaction [17, 91]. Multiplex qPCR further expands diagnostic capability by simultaneously detecting BFDV, avian polyomavirus (APV), and PsHV, making it particularly useful for evaluating immunocompromised birds presenting with overlapping clinical syndromes [92].

Genetic diversity among circulating BFDV strains continues to present a significant diagnostic challenge. Field isolates may differ substantially from reference genomes. For example, highly divergent BFDV variants detected in rainbow bee-eaters (*Merops ornatus*) required degenerate primer sets for successful amplification [24]. Furthermore, individual hosts may harbor multiple viral variants exhibiting considerable sequence divergence within the *rep* gene [15]. These findings emphasize the importance of targeting conserved genomic regions, incorporating degenerate or multiplex primer designs, and confirming suspected divergent strains using HRM analysis or nucleotide sequencing [36].

LAMP represents an attractive alternative for resource-limited laboratories because amplification occurs under isothermal conditions without the need for a conventional thermal cycler. A validated swarm-primer LAMP (sLAMP) assay demonstrated a detection limit of 5 × 10² viral copies per reaction [5]. Consequently, LAMP is particularly well suited for rapid diagnostic triage in situations where immediate screening is prioritized over detailed phylogenetic characterization, such as preliminary quarantine assessments. In contrast, metagenomic next-generation sequencing (NGS) provides the highest level of genomic resolution by enabling recovery of highly divergent viral strains and simultaneous detection of co-infecting pathogens that targeted molecular assays may fail to identify. However, high costs, specialized laboratory infrastructure, and prolonged analytical turnaround currently restrict its routine application (Table 3).

Despite these advances, the deployment of point-of-care molecular assays for BFDV remains only partially validated under field conditions. The sLAMP assay targeting ORF V1 amplifies viral DNA at 62°C within approximately 40 min and permits naked-eye interpretation of results through colorimetric detection. Using clinical psittacine samples, this assay demonstrated a detection limit of 5 × 10² DNA copies per reaction and achieved complete agreement with qPCR, with both methods identifying 36.0% of samples as positive (100.0% concordance; κ = 1.0). By comparison, an earlier LAMP assay detected only 25.6% of the same clinical samples [5]. Comparable field-adjacent applications of LAMP include surveillance for fowl adenovirus D (FAdV-D) in wild birds (8/317 positive; 2.52%), trace DNA detection from invasive reptiles within 30 min at concentrations as low as 1 fg, and *Leptospira* surveillance in wild rodents, which demonstrated 9.0% positivity and substantial agreement with PCR (κ = 0.77) [93–96]. Collectively, these findings support the use of LAMP as a rapid diagnostic platform in laboratories with limited resources. However, current evidence remains insufficient to confirm reliable performance under true field conditions, including robustness to crude sample extraction, cold-chain-independent workflows, PCR inhibitors, or operator variability at wildlife capture sites, quarantine facilities, or nest-box surveillance programs. Likewise, HRM analysis is best regarded as a rapid genotyping and diagnostic triage tool. Although it can discriminate known BFDV genotypes following PCR amplification, it still requires an HRM-compatible real-time PCR instrument together with validated reference melting profiles. Consequently, HRM should not be considered a true cage-side diagnostic method [36].

¹Analytical limits of detection (LOD) represent values reported for representative assays under optimized laboratory conditions (copies/reaction where specified). Performance characteristics reported in different studies are not directly comparable and should not be interpreted as equivalent measures of clinical sensitivity or specificity. Diagnostic sensitivity in naturally collected specimens may be reduced by PCR inhibitors, including melanin, lipids, bile salts, and hemoglobin.

**Table 3:** Comparison of molecular diagnostic methods for Beak and feather disease virus (BFDV).

**Method**	**Analytical LOD (copies/reaction)**	**Reported performance**	**Primary diagnostic value**	**Workflow requirements and application**	**Limitations/references¹**
Conventional PCR	13 (comparative assay)	Universal ORF1 assay successfully amplified samples from birds with PBFD lesions	Initial molecular screening	Thermal cycler; suitable for flock- or facility-level surveillance	Analytical sensitivity depends on assay design; primer selection influences detection of divergent strains [36, 90]
TaqMan qPCR	10	Limit of detection = 10; limit of quantification = 30 in a 2025 assay; broad variant detection demonstrated in a 2017 assay	Viral load quantification and diagnostic confirmation	Real-time PCR platform; quarantine screening, clinical monitoring, and eDNA verification	Requires calibrated quantitative standards and laboratory infrastructure [17, 91, 97]
Triplex qPCR	<6	100% analytical specificity; confirmed 98% of histopathology-positive cases	Simultaneous differential diagnosis of multiple pathogens	Real-time PCR platform; concurrent detection of BFDV, APV, and PsHV	Restricted to targeted pathogens; stringent contamination control required [92]
LAMP/sLAMP	5 × 10²	Complete agreement with qPCR (100% concordance) in evaluated clinical samples	Rapid diagnostic triage in resource-limited settings	Heat block with colorimetric readout; preliminary quarantine screening	Performance under field conditions and tolerance to sample inhibitors remain incompletely validated [5]
HRM analysis	13–48	HRM-*cap* assay specificity of 99.9%; κ = 0.87 compared with conventional PCR	Rapid genotype discrimination	HRM-compatible real-time PCR platform; variant surveillance	Requires validated reference melt profiles and specialized analytical software [36]
NGS/metagenomics	Variable	Performance depends on sequencing workflow and genome coverage	Whole-genome surveillance, variant discovery, and co-infection detection	High-throughput sequencing platform; outbreak investigation and molecular epidemiology	Expensive, bioinformatically intensive, and unsuitable for routine diagnostic triage [98]

### Sample selection and serological approaches

Selection of the appropriate sample type has become a major determinant of diagnostic sensitivity because blood, feathers, skin, cloacal or fecal swabs, and environmental specimens reflect different stages of viral replication, tissue persistence, shedding, or environmental contamination. In experimentally and clinically infected budgerigars, Hess *et al.* [64] reported PCR positivity rates of 67% for feather samples, 45% for cloacal swabs, and only 21% for blood samples. Similar findings have been reported in crimson rosellas, in which blood-negative but tissue-positive infections were common, while combined testing of skin and blood feathers substantially improved diagnostic sensitivity [7]. Consequently, optimal surveillance strategies should employ sample matrixes according to the specific diagnostic objective, whether acute clinical diagnosis, quarantine-release, flock surveillance, or environmental monitoring.

Environmental surveillance likewise requires standardized sampling procedures. The most thoroughly validated field protocol consists of systematic nest-box swabbing before, during, and after the breeding season, together with matched control samples collected from nest boxes occupied by BFDV-negative birds and from unoccupied nest boxes [8]. Sampling should include the inner nest-box walls, entrance surfaces, nesting material, accumulated feather dust, and fecal debris, followed by nucleic acid extraction incorporating appropriate inhibition controls because environmental dust and fecal material frequently contain substances capable of suppressing PCR amplification. Nest-box eDNA surveillance provides an effective, non-invasive method for colony-level risk assessment and evaluation of cleaning or decontamination procedures. Nevertheless, detection of BFDV DNA in environmental samples should be interpreted as evidence of environmental contamination or previous exposure rather than proof of active infection in an individual bird or the presence of infectious virions.

**Figure 6:**
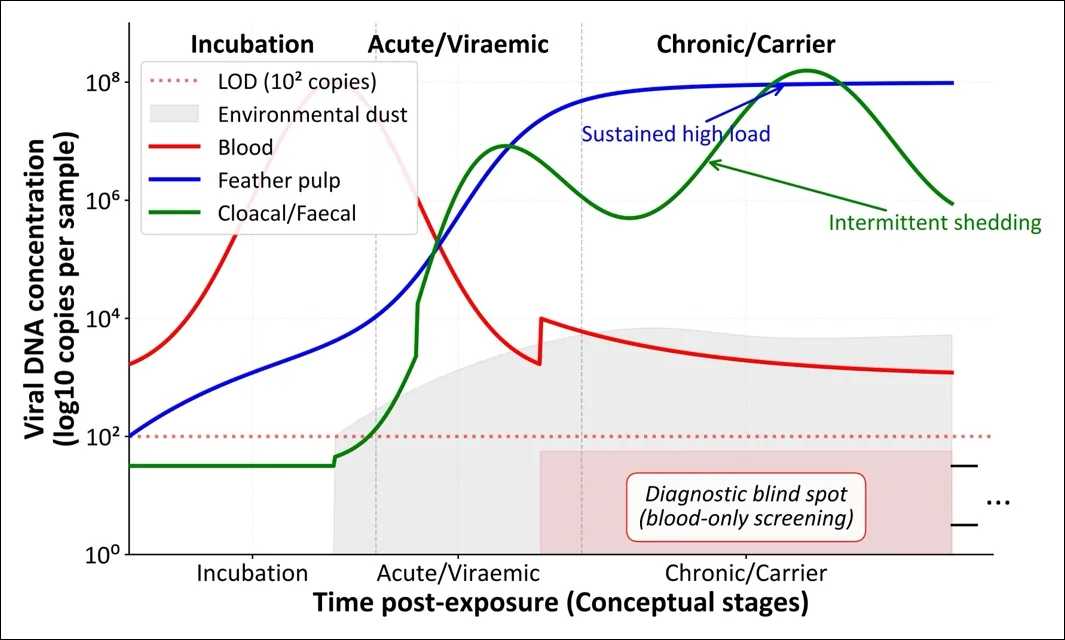
Spatiotemporal dynamics of Beak and feather disease virus detection sensitivity across diagnostic specimen types. The conceptual model illustrates viral load trajectories in blood (red), feathers (blue), swabs (green), and environmental dust (gray). Vertical dashed lines delineate successive stages of infection, whereas the horizontal dotted line indicates the analytical limit of detection (10² viral copies). The shaded red region represents a diagnostic blind spot in which blood-only screening may fail because of tissue compartmentalization. Curves integrate evidence from studies reporting persistent tissue infection despite blood negativity [7], intermittent viral shedding (56%) [21], nest-box positivity during breeding (36.4%), and environmental DNA persistence for up to 3.7 months after nest occupancy [8]. This model represents a conceptual synthesis across multiple psittacine species; individual infection dynamics may vary according to host species, age, and environmental conditions.

Serological methods have advanced considerably during the past decade, although interpretation remains highly dependent on clinical and epidemiological context. Early HI assays were constrained by variability in reagent quality and differences among specimen types [59]. More recent antigen-capture ELISAs permit direct detection of viral proteins [99], whereas recombinant VLP-based iELISAs detect anti-BFDV antibodies with high analytical sensitivity and strong agreement with HI assays [63]. Plant-based recombinant expression systems, particularly *Nicotiana benthamiana*, offer a cost-effective platform for producing recombinant Cap antigens for both serodiagnostic assays and candidate vaccine development [20, 38]. Collectively, these technologies strengthen population-level serosurveillance and assessment of viral exposure. However, serological results alone should not be interpreted as evidence of active infection, viral clearance, or protective immunity (Figure 6).

Emerging molecular technologies should be regarded as complementary surveillance tools rather than replacements for validated qPCR assays. Diagnostic multiplexing has already progressed beyond single-pathogen detection. Triplex qPCR assays simultaneously detect BFDV, avian polyomavirus (APV), and PsHV within a single reaction [92]. More recently, a companion-bird diagnostic workflow validated multiplex qPCR for BFDV, APV-1, and PsHV-1, demonstrating an analytical sensitivity of 10 copies/μL for each target and an estimated BFDV amplification efficiency of 97.54% [61]. Metagenomic sequencing is particularly valuable when syndromic disease investigations, wildlife trade surveillance, or outbreak investigations require unbiased detection of multiple pathogens. For example, fecal virome sequencing of clinically healthy monk parakeets identified viruses belonging to the families Circoviridae, Parvoviridae, and Adenoviridae, including BFDV, within a single sequencing workflow [98]. In contrast, no validated BFDV-specific Clustered regularly interspaced short palindromic repeats (CRISPR)-Cas diagnostic platform or portable nanopore sequencing workflow was identified during the literature review. These technologies therefore represent important priorities for future research rather than evidence-based diagnostic tools currently suitable for routine application.

An additional limitation affecting current molecular diagnostics is the lack of inter-laboratory standardization. Published protocols differ considerably in sample type, nucleic acid extraction methods, primer and probe design, target genomic regions, quantification cycle (Cq) thresholds, sample storage conditions, inhibition controls, and confirmatory procedures such as HRM analysis or sequencing [17, 36]. To improve comparability among laboratories, minimum reporting standards should include detailed descriptions of these methodological variables together with quantitative copy-number calibration or clearly defined qualitative interpretation criteria. Reports should also specify the viral genotype used as the positive control. External proficiency testing panels should incorporate genetically divergent BFDV strains, low-copy-number reference materials near the analytical limit of detection, and inhibitor-spiked matrixes prepared from feathers, feces, blood, and environmental samples. Without internationally harmonized reference materials and reporting standards, prevalence estimates and trade certification data may appear quantitative while remaining difficult to compare across laboratories and geographical regions.

**Figure 7:**
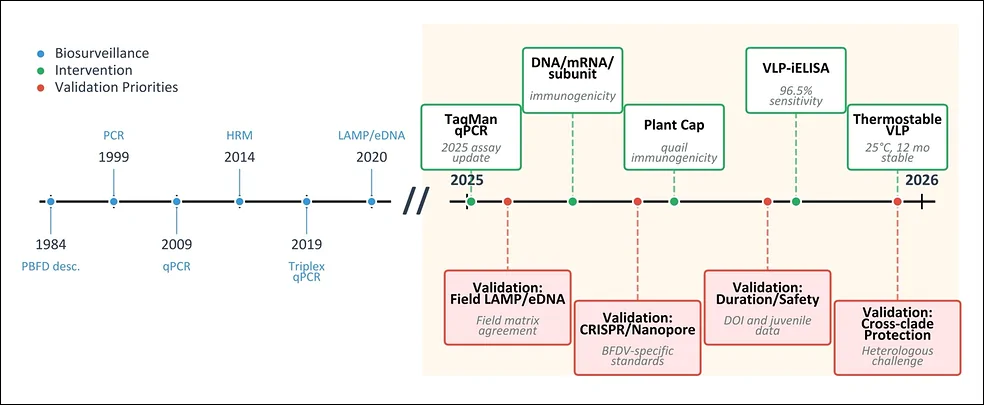
Timeline illustrating the development of BFDV diagnostics and vaccine research (1984–2026). The diagnostic pathway extends from the original pathological description of PBFD [50] through the introduction of universal PCR assays [90], qPCR and TaqMan viral load quantification [17, 91, 97], triplex and multiplex molecular diagnostics [61, 92], HRM-based genotyping [36, 100], nest-box eDNA surveillance [8], and VLP-based iELISA serodiagnostic platforms [63]. The vaccine development pathway summarizes DNA-, messenger RNA-, and subunit vaccine immunogenicity studies [18], plant-produced recombinant Cap vaccine platforms [20], and thermostable spray-dried VLP formulations [19]. The final panel highlights major research priorities, including field validation of LAMP assays [5], development of BFDV-specific CRISPR diagnostics, standardized portable sequencing workflows, duration of immunity studies, evaluation of safety in juvenile birds, and assessment of cross-genotype protection and target-species safety.

These challenges in diagnostic standardization define the transition from laboratory detection to practical disease intervention and are summarized in the diagnostic-vaccine timeline presented in Figure 7. Despite substantial advances in assay development, a significant gap remains between published laboratory methodologies and validated field applications. Consequently, many technological innovations have yet to be translated into standardized operational practice. LAMP-based diagnostic workflows still require comprehensive field validation, whereas BFDV-specific CRISPR assays and standardized portable sequencing protocols remain unavailable or insufficiently validated. Likewise, critical information regarding duration of immunity, safety in juvenile birds, cross-genotype protection, and target-species safety is still lacking for candidate vaccines, representing essential knowledge gaps before widespread field implementation can be recommended.

### Diagnostic entry points for the framework

Recent advances in BFDV diagnostics provide the foundation for assigning birds, facilities, and habitats to the appropriate components of the proposed BFDV Resilience Framework. qPCR generates quantitative evidence of viral burden [17, 91, 97], whereas HRM analysis or nucleotide sequencing provides information on genotype-associated risk and viral diversity [36]. Serological assays contribute complementary evidence regarding previous exposure and immune history [59, 63], while environmental qPCR and eDNA surveillance characterize site-level contamination and environmental persistence [8]. Consequently, diagnostic interpretation should consider both the specimen type and the strength of supporting evidence. Positive results obtained from feathers, feces, or environmental samples have different epidemiological implications from isolated blood positivity, whereas a negative result obtained from a single sample matrix should not be regarded as sufficient to exclude infection in high risk birds or populations [7, 21].

## PREVENTION AND CONTROL STRATEGIES

This section translates the proposed three-pillar BFDV Resilience Framework (Figure 1) into a practical, tiered biosecurity strategy. Within this framework, surveillance establishes the level of epidemiological risk, intervention reduces viral exposure and disease associated welfare impacts, and ecological monitoring continuously updates management decisions relating to wildlife trade, conservation translocations, and future vaccination strategies.

### Operationalizing the resilience framework

Implementation of the resilience framework follows a logical progression from surveillance to intervention. Active biosurveillance begins with qPCR testing supported by HRM analysis or sequence confirmation for genotype verification [17, 36]. When single-matrix testing provides insufficient diagnostic confidence, additional sampling of multiple tissues or environmental matrixes should be undertaken to improve sensitivity [7, 8]. Strategic intervention integrates quarantine, biosecurity measures that minimize viral exposure, environmental deconta-mination, and supportive clinical management where appropriate [23, 101]. Assessment of vaccine readiness should remain a separate component because current VLP-based and related vaccine platforms remain experimental or are supported only by limited historical evidence [19, 25]. The ecological and evolutionary monitoring component initially evaluates indicators of host-genomic resilience [26] before integrating these data with molecular surveillance of viral recombination and phylodynamic spread [16, 68]. These combined datasets subsequently inform wildlife trade policy, conservation translocation planning, and long-term disease management [89, 102].

To facilitate operational implementation, we propose a preliminary BFDV Operational Risk Score (BORS) as a structured decision-support tool. This scoring system should be regarded as a hypothesis-generating framework requiring prospective validation before application to management decisions. BORS assigns scores from 0 to 3 across six independent domains. Domain 1 evaluates infection burden using prevalence or repeated positive detections, with the pooled global prevalence estimate of 16.30% serving only as a contextual reference rather than a universal threshold [71]. Domain 2 assesses viral burden and specimen evidence, ranging from negative multi-matrix testing to high viral loads or concurrent positivity in multiple tissues, feathers, feces, or environ-mental samples. Domain 3 evaluates host conservation value and biological susceptibility, with critically endangered species, conservation translocation programs, neonates, and immunologically naïve populations receiving the highest scores [45, 73]. Domain 4 measures genomic risk based on detection of recombinant genomes, highly divergent variants, or previously undescribed phylogenetic lineages. Domain 5 assesses trade-associated connectivity, including commercial bird movements, mixed-species exhibitions, import-export interfaces, and online wildlife trade networks [61, 72]. Finally, Domain 6 evaluates uncertainty surrounding host resilience, including reduced or unknown MHC and TLR diversity, low host heterozygosity, genetically rare populations, or documented disease-associated population declines [60].

Under this proposed framework, total BORS values of 0–3 indicate low epidemiological risk, for which routine surveillance and standard hygiene practices are considered appropriate, consistent with existing recommend-dations for disease prevention in managed bird populations [23]. Scores of 4–7 indicate moderate risk and warrant enhanced surveillance, including repeated multi-matrix qPCR testing and environmental sampling. Scores of 8–12 indicate high risk and should trigger temporary movement restrictions, facility biosecurity audits, HRM or sequence confirmation, and targeted escalation of control measures when diagnostic uncertainty or novel viral genotypes are identified. Scores of 13–18 represent outbreak or conservation-emergency situations requiring quarantine, contact tracing, environmental decontamination, and suspension of bird movements or conservation releases. Investigational vaccination should only be considered following ethical approval and when regulatory and safety requirements have been satisfied. These proposed score categories represent decision-support guidance rather than universally validated cutoff values. Thresholds should therefore be recalibrated according to host species, sampling strategy, viral copy-number calibration, diagnostic assay performance, welfare considerations, and local epidemiological conditions.

The resulting decision tree is intentionally precautionary. In low-prevalence wild populations without clinical disease and with only sporadic environmental detections, management should emphasize non-invasive eDNA surveillance, nest-site hygiene, habitat management, and seasonal resampling. Captive breeding establishments and commercial bird facilities with repeated positive detections, multispecies housing, or genetically divergent viral strains should implement entry quarantine, species segregation, environmental decontamination, repeat qPCR surveillance, and sequence-informed movement restrictions. Within conservation translocation programs, detection of BFDV in source, release, or post-release populations should prompt temporary suspension of releases until comprehensive reassessment of released birds, nesting sites, and sympatric wild populations has been completed. In threatened populations, clinical PBFD, high viral loads, recombinant viral genomes, or repeated multi-matrix positivity should initiate emergency quarantine, contact tracing, environmental surveillance, welfare assessment, and formal consideration of investigational vaccination or other interventions under appropriate ethical and regulatory oversight. Accordingly, Figure 1 should be regarded not merely as a conceptual overview but as an operational decision algorithm linking risk classification to evidence-based management actions.

### Vaccine development

As of 2026, no licensed commercial vaccine or antiviral therapy is available for BFDV infection [2]. Most vaccine investigations remain at the proof-of-concept or preclinical stage, and no vaccine has yet received regulatory approval for routine field use [18–20]. The strongest evidence for protective efficacy continues to derive from early challenge studies using experimental inactivated whole-virus vaccines. Adult and nestling cockatoos vaccinated with a double-oil emulsion vaccine developed detectable antibody responses, and vaccinated nestlings were protected following experimental challenge. In contrast, unvaccinated control chicks developed acute PBFD within four weeks of challenge. However, three vaccinated wild caught sulfur-crested cockatoos subsequently died of PBFD before challenge despite possessing detectable antibody responses [25]. These findings illustrate that induction of measurable antibodies does not necessarily equate to clinical protection, particularly when vaccination occurs during the incubation period or when host age, species, immune competence, and infectious challenge differ.

Recent vaccine candidates reported during 2025–2026 represent important advances but should be interpreted cautiously. Ndlovu *et al.* [18] vaccinated 10-week-old African gray parrot chicks using three experimental platforms: a plant-produced BFDV coat protein (Cap) subunit vaccine (20 μg), a tobacco mosaic virus (TMV)-encapsidated messenger RNA (mRNA) vaccine (100 μg), and a BFDV 1.1-mer DNA vaccine (90 μg). Birds received two booster vaccinations at 2-week intervals. All three vaccine platforms elicited anti-BFDV Cap antibody responses by day 42, with the recombinant subunit vaccine producing the highest binding antibody titers (>6400). Nevertheless, the study did not include live-virus challenge, viral shedding measurements, tissue viral load quantification, assessment of immunity beyond day 42, or heterologous genotype challenge. Similarly, plant-produced recombinant BFDV Cap expressed in *Nicotiana benthamiana* induced serum and egg-yolk immuno-globulin Y (IgY) responses in Japanese quail, although antigen yield remained relatively low (1.58 mg/kg fresh biomass), and protective efficacy against PBFD was not evaluated [20].

Among recently reported vaccine technologies, the spray-dried VLP formulation described in 2026 represents the most practically significant advance because it addresses vaccine stability and field deployment rather than antigen design alone. This platform builds upon previous demonstrations that plant-expressed BFDV Cap proteins self-assemble into VLPs [38]. Das *et al.* [19] optimized spray drying to produce particles measuring 3.1–9.5 μm in diameter with residual moisture contents ranging from 1.7% to 8.6%. These particles retained hemagglutination (HA) antigenicity after exposure for 1 h across temperatures ranging from 4°C to 65°C and remained antigenically stable for more than 12 months at room temperature. In specific-pathogen-free (SPF) chickens, 100 μL of spray-dried VLP suspension administered on days 3, 16, and 30 induced anti-BFDV Cap antibody titers of 51,200 on day 30 and 409,600 on day 44 following intramuscular administration. By comparison, the oil-adjuvanted intramuscular formulation achieved titers of 102,400 on day 44, whereas the oculonasal spray-dried formulation produced titers of only 6,400. Intracloacal administration failed to induce detectable antibody responses. Despite these encouraging formulation data, protective efficacy following experimental BFDV challenge, evaluation in psittacine target-species, cross-genotype protection, and neonatal safety studies remain unavailable. Consequently, although spray drying substantially improves vaccine stability, critical questions regarding protective efficacy and optimal delivery routes remain unresolved.

Cross-genotype protection represents the most significant unresolved challenge in BFDV vaccine development. Most current vaccine candidates target the Cap protein, whereas complete-genome analyses reveal extensive phylogenetic divergence and frequent recombination among circulating BFDV strains [16]. At present, standardized virus neutralization assays, antigenic cartography, and homologous-versus-heterologous challenge studies have not been established, preventing robust evaluation of cross-clade vaccine efficacy. Conservation considerations introduce additional constraints because placebo-controlled challenge studies are generally unacceptable in endangered psittacine species. Consequently, any future emergency use of investigational vaccines should require rigorous disease risk assessment, demonstrated batch potency, systematic adverse-event surveillance, post-release pharmacovigilance, and oversight within conservation translocation programs [102].

Selection of an appropriate vaccine delivery route represents an equally important translational challenge. Intramuscular administration generated the strongest immune responses in the 2026 VLP study, whereas oculonasal delivery produced substantially lower antibody titers and intracloacal administration failed to induce measurable responses [19]. Although repeated capture and intramuscular vaccination may be feasible within captive breeding programs, these approaches are considerably less practical for free-ranging parrots, particularly endangered or reintroduced populations, where repeated handling and capture-related stress represent substantial welfare concerns [23]. Alternative delivery approaches, including oral formulations, aerosol vaccination, nest-site application, or bait-based immunization, remain largely unexplored and will require independent evaluation of vaccine uptake, mucosal immunity, non-target exposure, environmental safety, and regulatory acceptability.

To avoid overstating the current evidence, antibody binding assays, HA or HI inhibition, and absence of short-term clinical disease should not be interpreted as evidence of sterilizing immunity. The 2025–2026 DNA, mRNA, plant-derived, and VLP-based vaccine platforms should therefore be regarded primarily as advances in vaccine formulation and immunogenicity rather than demonstrations of protective efficacy. Robust evidence from live-virus challenge studies, heterologous genotype challenge experiments, tissue viral load assessment, viral shedding analyses, duration of immunity studies, and target-species safety evaluations remains essential before these candidate vaccines can be considered for practical disease control (Table 4). This evidence gap directly reflects the extensive Cap diversity and ongoing viral recombination described earlier in this review and highlights the continuing need for comprehensive cross-clade efficacy studies before vaccination can become an evidence-based component of BFDV control.

**Table 4:** Expanded critical appraisal of current Beak and feather disease virus (BFDV) vaccine candidates.

**Platform**	**Model and vaccination schedule**	**Main outcome**	**DOI**	**Juvenile/neonatal evaluation**	**Cross-genotype data**	**Developmental stage and limitations**
BFDV 1.1-mer DNA vaccine	African gray parrot chicks; 90 µg SC on days 1, 14, and 28	Induced anti-BFDV Cap antibodies; no challenge study	Not evaluated	Small juvenile cohort	None	Preclinical; small sample size [18]
Inactivated double-oil emulsion vaccine	Adult and nestling cockatoos	Vaccinated nestlings were protected against challenge, whereas controls developed acute PBFD	Not defined	Three vaccinated wild caught birds died before challenge	None	Historical, unlicensed platform; limited sample size and uncertain pre-existing infection status [25]
Plant-produced Cap subunit vaccine	African gray parrot chicks; 20 µg SC on days 1, 14, and 28	Produced the highest antibody titer (>6400) among the three contemporary candidates	Evaluated only to day 42	Five birds/group; no neonatal evaluation	None	Preclinical; no challenge, shedding, or tissue viral load endpoints [18]
TMV-encapsidated mRNA vaccine	African gray parrot chicks; 100 µg SC on days 1, 14, and 28	Induced anti-BFDV Cap antibodies, but titers were lower than those induced by the Cap subunit vaccine	Evaluated only to day 42	No neonatal data	None	Preclinical; delivery, durability, and protective efficacy remain unresolved [18]
Spray-dried VLP vaccine	Day-old SPF chickens; administered on days 3, 16, and 30	High antibody titers after IM administration; weak or absent responses through mucosal routes	Immune DOI not evaluated; antigenicity retained for >12 months at RT	No evaluation in psittacine neonates	None	Thermostable formulation; no BFDV challenge or target-species efficacy data [19]

DOI = Duration of immunity, SC = Subcutaneous, TMV = Tobacco mosaic virus, mRNA = Messenger RNA, VLP = Virus-like particle, SPF = Specific-pathogen-free, IM = Intramuscular, RT = Room temperature.

In the absence of a licensed vaccine, BFDV containment depends on layered biosecurity rather than isolated control measures. Effective aviary protocols should integrate pre-entry screening, quarantine, segregation of birds according to age and infection risk, species-specific equipment, unidirectional staff movement, and mechanical removal of feather dust and organic debris before disinfection [23, 101]. Surveillance should additionally include longitudinal multi-matrix qPCR testing and regular environmental audits. For endangered or high-value species, sampling should include feathers, tissues where clinically justified, fecal or cloacal specimens, nest-box surfaces, and accumulated feather dust [7, 8]. These measures involve substantial costs related to labor, consumables, isolation facilities, environmental cleaning, and repeated diagnostic testing. However, formal cost-effectiveness comparisons among testing-only, sanitation-centered, depopulation, and integrated control strategies remain unavailable for PBFD.

The most rigorous field evaluation conducted to date illustrates the practical trade-offs associated with intensive biosecurity. In wild Mauritius echo parakeets (*Psittacula eques*), stringent nest-site biosecurity reduced the probability of infection among nestlings by approximately 11%. However, the intervention did not reduce individual viral loads or improve body condition. Fledging success was also slightly lower in treated nests than in untreated nests (79% vs. 83%) [23]. These findings indicate that the effectiveness of biosecurity interventions should be assessed using population-level conservation and reproductive outcomes rather than molecular clearance alone.

Chemical decontamination should begin with thorough mechanical removal of organic material, feather dust, feces, and contaminated nesting debris because these materials can sustain environmental exposure and reduce disinfectant efficacy [8, 101]. Direct evidence regarding the virucidal activity of disinfectants against BFDV remains limited, and many current recommendations are therefore extrapolated from studies of PCV2 [103, 104]. Peroxygen disinfectants, including potassium peroxymonosulfate, represent practical first-line options for routine facility use. Sodium hypochlorite may also be effective on non-porous surfaces but requires fresh preparation and is susceptible to inactivation by organic material, in addition to causing corrosion. Strong alkaline disinfectants should be restricted to empty facilities or controlled equipment-decontamination procedures because of their potential risks to birds, personnel, and treated materials.

Non-vaccine interventions should prioritize preservation of host genetic resilience while reducing opportunities for exposure. Selective breeding programs should not restrict endangered populations to a small number of birds presumed to possess resistant genotypes, as this could further reduce genetic diversity and adaptive capacity. Instead, managed breeding programs should preserve immune-gene diversity and use MHC and TLR data to inform surveillance intensity and pairing strategies rather than exclusion or culling. Current evidence remains associative. Red-crowned parakeets exhibited temporal changes in BFDV prevalence alongside immunity-associated variation at *TLR3* [60]. Hybrid crimson rosellas had lower infection prevalence and viral loads than their parental subspecies [88]. In addition, within the crimson rosella species complex, greater host heterozygosity or genotype rarity was associated with a reduced probability of infection or lower viral burden [26]. Functional validation is required before any of these genetic markers can be incorporated into breeding or conservation decisions.

Habitat and husbandry interventions should similarly aim to reduce repeated and concentrated exposure. Practical measures include limiting artificial aggregation, routinely rotating, cleaning, and disinfecting nest boxes, preventing contact between wild birds and captive or invasive psittacines, and restoring suitable nesting resources within conservation and reintroduction landscapes [2, 8, 12]. Supplemental feeding programs require particular caution because feeding stations may increase bird density and facilitate indirect transmission. In Mauritius echo parakeets, the adverse effect of a BFDV outbreak on hatching success was temporary but was disproportionately associated with breeding pairs receiving supplemental food [74]. Supplemental feeding should therefore use dispersed feeding points, frequent cleaning and disinfection, reduced crowding, and concurrent molecular surveillance to minimize transmission risk.

### Treatment and supportive care

As of 2026, no curative antiviral therapy or licensed treatment protocol is available for BFDV infection [43]. Consequently, clinical management remains supportive and welfare-oriented, with the primary objectives of minimizing mortality associated with dehydration, malnutrition, thermal stress, traumatic beak and feather lesions, and secondary opportunistic infections during circovirus-induced immunosuppression [45, 53]. Because current treatments do not reliably eliminate infection, every affected bird should be managed not only as an individual welfare case but also as a potential source of infection for aviaries, rehabilitation centers, and conservation release programs [101].

An evidence-based supportive care protocol should incorporate immediate isolation under barrier-nursing conditions, minimization of handling-related stress, provision of clean, warm, low-dust housing, individualized nutritional support, and fluid therapy guided by body weight, hydration status, crop function, and fecal output [101]. Feather dust, feces, and keratin debris should be removed each day mechanically before chemical disinfection because environmental contamination contributes substantially to ongoing viral exposure [8]. Clinical monitoring should include serial measurements of body weight, appetite, hydration, and hematological and biochemical parameters where available; assessment of feather and beak lesion progression; and repeated qPCR testing of multiple specimen types rather than blood alone. This approach is particularly important because clinically normal birds may harbor persistent tissue infection and exhibit intermittent viral shedding despite inconsistent detection in individual blood samples [7, 21].

Recognition and management of secondary infections represent a critical component of supportive care. Severe juvenile PBFD has been associated with lymphoid depletion and leukopenia, predisposing affected birds to opportunistic infections, including cryptosporidiosis in cockatoos [45, 48, 53]. Diagnostic evaluation should therefore be directed by clinical findings and may include fecal parasitological examination, cytology, bacterial or fungal culture, and molecular diagnostic testing when available. Antimicrobial, antifungal, antiparasitic, and anti-inflammatory therapies should be prescribed only when supported by compatible clinical, hematological, or laboratory evidence rather than administered empirically. This evidence-based approach is particularly important in conservation breeding programs because unnecessary treatment may compromise welfare, increase handling stress, and obscure disease investigations.

Prognosis depends on host age, species, immune status, viral burden, and severity of clinical lesions. Peracute disease in juvenile African gray parrots and systemic infections accompanied by leukopenia or hepatic necrosis are generally associated with a guarded to poor prognosis [45, 54]. Birds with chronic PBFD may survive for prolonged periods but frequently require long-term supportive management, including beak maintenance, feather-damage management, environmental modification, and regular quality-of-life assessments. Humane euthanasia may be justified when birds develop uncontrollable secondary infections, are unable to feed because of severe beak deformities, experience progressive debilitation, or present an unacceptable transmission risk to endangered or immunologically naïve populations despite appropriate isolation measures [101].

### Economic and social impacts

The consequences of PBFD extend well beyond direct veterinary care and have substantial implications for aviculture, wildlife conservation, and international trade. Since 1975, more than 19 million parrots have been legally traded internationally, creating repeated opportunities for BFDV introduction into commercial breeding operations, companion-bird collections, and conservation programs [6]. Infection of valuable breeding birds or endangered assurance colonies may result in mortality, reduced reproductive output, prolonged quarantine, repeated molecular testing, facility decontamination, movement restrictions, increased labor requirements, and substantial costs associated with replacement breeding stock or genetic management. A global meta-analysis published in 2025, encompassing 16,901 parrots representing 30 species from 34 countries across six continents, reported a pooled molecular prevalence of 16.30%, demonstrating that BFDV represents a widespread rather than geographically restricted disease burden [71].

Because formal economic evaluations of PBFD remain scarce, a structured scoping-cost framework provides a more defensible approach than unsupported monetary estimates. Three major cost domains can be identified. The first comprises avicultural losses associated with mortality, removal of breeding birds, and movement restrictions. The second includes rehabilitation-center expenditures, encompassing quarantine duration, repeated multi-matrix qPCR testing, diagnostics for secondary infections, daily husbandry, environmental decontamination, and opportunity costs associated with isolation facilities [7, 8, 101]. The third consists of conservation opportunity costs, including delayed releases, suspended translocations, missed breeding seasons, reduced founder representation, and diversion of financial resources from habitat restoration toward emergency disease management [73].

The social impact of PBFD also presents important implementation challenges. Wildlife hospitals and rehabilitation centers may be forced to divert limited personnel and infrastructure from routine clinical care toward isolation, molecular testing, and intensive biosecurity measures [101]. Informal and online wildlife trade further complicate disease control because the origin, health status, quarantine history, and diagnostic documentation of traded birds are frequently unknown. A 2026 investigation of online parrot trading in Indonesia documented 283 Facebook posts advertising 861 individually identifiable parrots representing 22 species across 38 Facebook groups [72]. These findings support implementation of affordable pre-sale and pre-export testing together with transparent health certification systems. Public communication should therefore emphasize that PBFD control serves both animal welfare and conservation biosecurity objectives.

Operationally, BFDV management should focus on reducing rather than eliminating disease risk. Field biosecurity interventions can decrease infection probability but may not necessarily improve reproductive success or fledging outcomes [23]. Conservation release programs should therefore integrate habitat restoration with formal disease risk analyses before translocation [12, 102]. Program-specific additions, including host genetic risk assessment and non-invasive environmental surveillance, can then further refine management decisions. Investigational vaccination should only be considered after these foundational measures have been implemented [8, 26].

### VLP regulation and endangered species deployment

No commercial vaccine against PBFD is currently available [2]. Existing vaccine evidence remains confined to preclinical investigations or limited historical studies. Consequently, investigational vaccines should not be deployed in endangered species until comprehensive data regarding safety, batch potency, adverse-event monitoring, diagnostic compatibility, and post-release surveillance become available [19, 25]. These safeguards should be incorporated into formal disease risk analyses supporting conservation translocations [102]. Planned reintroduction of Spix's macaws illustrates this challenge. Although endangered recovery programs may urgently require measures that reduce infectious disease risk [12, 73], implementation of emergency vaccination without validated safety, efficacy, and monitoring criteria would currently lack sufficient scientific and regulatory justification.

### CITES/IUCN integration and trade losses

The CITES remains the principal international framework governing trade in threatened wildlife species [89]. However, possession of valid trade permits alone does not ensure standardized BFDV testing or disease-free certification. Existing wildlife disease risk analyses for conservation translocations emphasize transparent hazard identification, uncertainty assessment, risk management, and ongoing review [102], yet PBFD remains inconsistently addressed within many psittacine conservation action plans. Stronger policy integration is supported by three independent lines of evidence: the international movement of more than 19 million parrots since 1975 [6], phylodynamic analyses linking BFDV dissemination with international bird trade [16, 105], and increasing evidence that informal and online trade frequently obscures bird origin and health status [72]. Formal economic comparisons among pre-export testing, post-import quarantine, environmental DNA surveillance, facility decontamination, and future vaccination strategies remain unavailable.

Global PBFD control continues to be constrained by regulatory and standardization gaps. Current trade documentation verifies legal movement but does not confirm BFDV-negative status, specimen chain-of-custody, diagnostic methodology, viral genotype, quarantine duration, or environmental status of exporting facilities. A risk-based international trade policy should therefore integrate wildlife permits with standardized pathogen documentation, including pre-export and post-import molecular testing, genotype confirmation of positive samples, environmental surveillance of high risk breeding facilities, and independent verification of health certification. Enhanced surveillance should also prioritize recurrent online trade networks where traceability remains limited [8, 16].

### Technological foresight within the BFDV Resilience Framework

Several emerging technologies have the potential to strengthen future BFDV surveillance and control but currently require pathogen-specific validation before operational implementation. CRISPR-Cas diagnostic platforms could provide rapid confirmation following LAMP or recombinase polymerase amplification (RPA), as activation of Cas12a results in collateral cleavage of single-stranded DNA reporter molecules [106]. For BFDV, however, guide RNA performance must first be validated across genetically divergent viral lineages and inhibitor-rich clinical and environmental specimens. Consequently, CRISPR diagnostics should presently be regarded as complementary to, rather than replacements for, laboratory-based LAMP assays [5].

Portable nanopore sequencing likewise offers promise for rapid field-based genotype confirmation and detection of recombinant viral genomes, analogous to its successful application during Ebola virus surveillance [107]. Before adoption for BFDV diagnostics, standardized criteria for genome coverage, consensus sequence accuracy, and recombination detection pipelines must be established for quarantine and surveillance applications [16].

Drone-assisted eDNA sampling may expand non-invasive surveillance to otherwise inaccessible canopy nest sites. Current evidence, however, derives from biodiversity monitoring rather than infectious disease investigations [108]. Application to BFDV surveillance will require validation of sampling efficiency, contamination control procedures, and environmental stability under varying climatic conditions using established nest-box eDNA methodologies [8]. Similarly, multivalent and nanoparticle-based VLP vaccines capable of incorporating both conserved and clade-specific Cap epitopes represent scientifically plausible future strategies, but these concepts remain hypothetical until supported by target-species immunogenicity studies and heterologous challenge experiments [18, 19].

Finally, artificial intelligence-assisted phylodynamic forecasting could eventually integrate viral genomic data, wildlife trade connectivity, and global prevalence trends, analogous to international influenza and SARS-CoV-2 surveillance platforms such as GISAID and Nextstrain [109, 110]. Such systems would require a curated, open-access BFDV genomic database containing standardized metadata and should initially function as validated decision-support tools for surveillance prioritization and trade-risk assessment rather than autonomous management systems [16].

### Operational response tiers

Prevention and control of BFDV should follow a structured, tiered response framework rather than relying on isolated interventions. Low-risk situations primarily require routine surveillance, hygiene, and continued monitoring [23]. Commercial trade facilities and captive breeding collections require additional measures, including quarantine, species segregation, environmental verification, and sequence-based surveillance of viral diversity [8, 16]. Conservation translocation programs should incorporate temporary release suspension when necessary together with comprehensive post-release surveillance within a formal disease risk assessment framework [102]. Where available, host-genomic information should provide an additional layer of risk stratification [26]. During outbreaks affecting endangered populations, management may require emergency biosecurity, intensive welfare assessment, and carefully reviewed consideration of investigational vaccination. Nevertheless, current vaccine platforms remain preclinical, and historical evidence of protection remains limited; consequently, vaccination should not replace established surveillance and biosecurity measures until robust efficacy and safety data become available [18, 19, 25].

## FUTURE PERSPECTIVES AND RESEARCH PRIORITIES

BFDV control now encompasses viral evolution, global commerce, and escalating conservation risks for psittacine species. Four major translational barriers remain particularly important. First, validated field interventions and transmission models for free-ranging populations are still limited. Intermittent viral shedding and prolonged environmental persistence complicate decisions regarding infection clearance [8, 21], and hygiene-based interventions do not necessarily translate into improved fledging success [23]. Second, cross-species detections require mechanistic confirmation before they can be interpreted as evidence of spillover events or reservoir establishment [13, 14]. Third, although current vaccine candidates have shown encouraging immunogenicity, none has demonstrated cross-genotype protection despite extensive genomic diversity and frequent recombination revealed by complete-genome analyses [16]. Furthermore, recent vaccine studies continue to rely primarily on injectable delivery systems rather than validated low-handling approaches suitable for free-ranging or conservation-dependent populations [18, 19]. Fourth, global surveillance remains constrained by assay heterogeneity and the absence of shared reference materials, inter-laboratory proficiency testing, and standardized minimum reporting criteria for molecular diagnostics [17, 36]. The sections below outline key priorities for surveillance, host-resilience research, diagnostic harmonization, vaccine development, and governance that address these critical barriers.

### Understanding subclinical infection dynamics

Subclinical infection remains one of the most important unresolved barriers to effective BFDV control. Current evidence demonstrates persistent tissue infection in clinically normal parrots [7] together with intermittent viral shedding in wild populations [21]. Consequently, surveillance programs should incorporate repeated multi-matrix sampling and environmental monitoring rather than relying solely on a single clinical examination or blood sample.

### Characterizing the mechanisms of cross-species transmission

BFDV is primarily a pathogen of psittacine birds; however, molecular detections in other avian orders require careful interpretation. Rainbow bee-eaters provide evidence consistent with a self-limiting cross-order infection event [24]. In contrast, detections in red goshawks may represent exposure through predation on infected psittacines or, alternatively, genuine infection [13]. Although reports involving passerines and pigeons broaden the spectrum of non-psittacine detections, these findings should not be interpreted as evidence of reservoir status without additional supporting data [83, 84]. Confirmation of reservoir status should require evidence of tissue replication, viral shedding, serological responses, repeated detection, and sustained conspecific transmission rather than PCR positivity alone.

### Elucidating the host determinants of resilience

**Figure 8:**
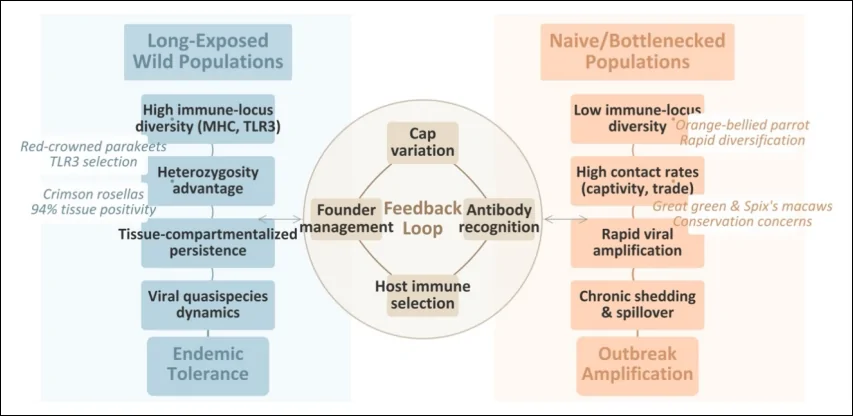
Co-evolutionary arms race model. Conceptual framework illustrating contrasting evolutionary outcomes in host populations. Left: Endemic tolerance, where long-term viral exposure may maintain immune-locus diversity, including major histocompatibility complex and *TLR3* variation [60], while tissue-compartmentalized viral persistence continues [7]. Right: Outbreak amplification, in which naive or genetically bottlenecked populations may experience rapid viral diversification [15] and chronic viral shedding, further intensified by high-density trade or captive management. The central loop represents bidirectional interactions between capsid evolution and host immune selection. Solid arrows indicate proposed causal pathways, whereas dashed arrows denote feedback mechanisms requiring validation through longitudinal studies.

Host-resilience likewise requires rigorous mechanistic investigation. Hybrid crimson rosellas have exhibited lower BFDV prevalence and viral loads than their parental lineages [88], whereas red-crowned parakeets demonstrated a temporal decline in BFDV prevalence associated with signatures of selection at *TLR3* [60]. The recently published crimson rosella genome provides an important resource for experimentally testing these associations [111]. Future studies should characterize MHC and *TLR* variation, investigate antiviral immune pathways, and use host-genomic information to guide founder pairing and preserve immune-gene diversity rather than promote narrow genetic selection or culling. Collectively, these observations support a host-virus co-evolutionary model (Figure 8).

### Advancing diagnostic harmonization

The molecular diagnostic repertoire for BFDV has expanded considerably, progressing from conventional PCR to HRM analysis, TaqMan qPCR, LAMP, and eDNA surveillance. Nevertheless, cross-border comparability remains limited because of differences in sample matrixes, nucleic acid extraction methods, primer and probe targets, Cq thresholds, and confirmatory procedures. Sample selection is particularly critical because birds that are negative in blood samples may remain positive in tissues [7], whereas primer selection and HRM assay design influence the detection of genetically divergent strains [36]. Quantitative interpretation also depends on appropriate assay calibration and clearly defined analytical detection thresholds [86]. Furthermore, BFDV populations comprise intra-host variants and mixed-variant infections [15, 47], while complete-genome analyses have demonstrated recurrent recombination events [16]. Consequently, future diagnostic development should prioritize broad-reactivity assays, standardized reference materials, inter-laboratory proficiency testing, and HRM analysis or sequencing to confirm genetically divergent positive samples.

### Accelerating vaccine translation and integrated biosecurity

Plant-based expression systems have enabled recombinant BFDV Cap production and the assembly of VLPs [38]. In addition, DNA, mRNA, subunit, and spray-dried VLP platforms have demonstrated encouraging immuno-genicity or formulation feasibility. However, these candidates have not yet generated evidence regarding homologous or heterologous challenge protection, duration of immunity, juvenile or neonatal safety, target-species safety, or validated low-handling delivery strategies [18, 19]. Historical challenge studies remain limited in both scale and scope [25]. Therefore, candidate vaccines should be evaluated as components of integrated disease control programs that combine surveillance, quarantine, habitat and nest management, environmental decontamination, and trade screening rather than as stand-alone interventions.

### Integrating molecular surveillance with conservation action

The proposed Genomic Intelligence Hub should serve as the operational component of the resilience framework by linking surveillance outputs with evidence-based intervention decisions while functioning as a decision-support platform rather than a universally validated algorithm. Its decision thresholds, sampling frequency, and response triggers should be calibrated according to species conservation status, diagnostic assay performance, local disease prevalence, animal welfare considerations, and regulatory requirements. Initial implementation should focus on four priority areas: (1) standardized diagnostics at wildlife trade hotspots; (2) environmental verification in high risk breeding and holding facilities; (3) non-invasive surveillance in free-ranging populations; and (4) carefully regulated vaccine trials conducted only under appropriate ethical and regulatory approval (Figure 1).

### Research gaps and prioritization matrix

The prioritization matrix (Table 5) categorizes major research gaps according to urgency, feasibility, and conservation impact, thereby providing a structured framework for allocating resources to operational BFDV control. It is intended as a synthesis tool that highlights research priorities and implementation value. Each research gap is linked to an appropriate study design, measurable outcome, and anticipated conservation benefit. Highly urgent and high-impact gaps should receive immediate investment, whereas research questions with high feasibility may generate early validation data that facilitate subsequent large-scale investigations.

**Table 5:** Research gaps and prioritization matrix for Beak and feather disease virus (BFDV) control.

**Topic/** **research gap**	**Urgency**	**Feasibility**	**Conservation impact**	**Evidence cue**	**Priority/endpoints**
Subclinical shedding	High	Moderate	High	Intermittent shedding and tissue persistence [7, 21]	Two- to 24-month longitudinal multi-matrix qPCR; shedding frequency; evidence-based quarantine-release criteria
Field vaccine efficacy	Very high	Low	Very high	Immunogenic vaccine platforms without heterologous challenge data [18, 19]	Challenge studies in surrogate psittacines; viremia, viral shedding, lesion severity, and breakthrough Cap mutations
Non-invasive eDNA	High	High	High	Persistence of nest-box DNA and shedding associated with breeding nests [8]	Validation of nest dust, feathers, and fecal eDNA; analytical LOD, inhibition, and environmental clearance thresholds
Trade and online markets	High	Moderate	High	Large legal trade volume and limited transparency in online markets [6, 72]	Trade-route risk modeling, estimates of untested bird movements, and cost-benefit analyses of diagnostic screening

LOD=Limit of detection, eDNA=Environmental DNA, qPCR=Quantitative polymerase chain reaction.

### Knowledge gaps summary linked to future perspectives

The priorities outlined in Table 5 identify three overarching research needs. First, prospective PBFD case registries should systematically record species, age, viral load, hematological findings, co-infections, treatment, survival, viral shedding, and quality-of-life outcomes because current clinical and rehabilitation evidence remains largely descriptive or based on individual case reports [45, 101]. Second, longitudinal multi-matrix qPCR studies are required to establish evidence-based retesting intervals, release criteria, and quarantine discontinuation protocols for birds exhibiting subclinical persistence and intermittent viral shedding [7, 21]. Environmental DNA studies should independently define clearance thresholds for both nest sites and captive facilities [8]. Third, implementation research should integrate diagnostic proficiency panels [38, 86], economic analyses of trade and online-market surveillance [6, 72], vaccine challenge endpoints for contemporary vaccine platforms [18, 19], and governance frameworks supporting wildlife trade and conservation translocations [89, 102]. Although historical challenge studies provide valuable preliminary evidence, they should not substitute for modern target-species evaluations and cross-genotype challenge experiments [25].

### Time-bound research and policy roadmap (2026–2035)

Future BFDV mitigation efforts should translate the identified knowledge gaps into measurable, time-bound deliverables rather than reiterating broad research priorities. Accordingly, the proposed roadmap is organized into progressive phases based on outcomes that can be implemented, evaluated, and refined.

**Phase 1: Standardization and framework drafting (2026–2027):** The first priority should be development of a harmonized diagnostic package comprising three key components: (1) standardized reporting requirements for sample matrixes and Cq values; (2) genotyped reference materials for inter-laboratory comparison of PCR, HRM, and qPCR assays; and (3) inhibitor-rich validation panels for field-oriented LAMP assays [5]. A second deliverable should be a standardized operating procedure for eDNA surveillance of nest boxes and facility dust, with clearance criteria evaluated across diverse ecological settings [8]. Collectively, these outputs would provide auditable inputs for national PBFD action plans, including prevalence baselines [71], wildlife rehabilitation biosecurity [101], and wildlife trade surveillance [72].

**Phase 2: Vaccinology and regulatory integration (2028–2030):** The principal objective of this phase should be development of a vaccine translation protocol that accounts for genetically divergent viral clades and recurrent recombination [16]. Contemporary DNA, mRNA, subunit, and spray-dried VLP platforms provide promising candidate technologies [18, 19], whereas historical challenge studies offer only limited benchmarks for protective efficacy [25]. The protocol should define standardized homologous and heterologous challenge endpoints, including viral load, shedding kinetics, lesion prevention, and breadth of serological responses. Concurrently, pilot BFDV health certification templates should be developed to align with CITES documentation requirements and wildlife disease risk analyses for conservation translocations [89, 102].

**Phase 3: Clinical deployment and global governance (2031–2035):** The final phase should evaluate vaccine safety and immunogenicity in target psittacine species after the successful completion of earlier challenge and monitoring milestones. Any adaptive or emergency use vaccination framework should adhere to established principles of wildlife disease risk assessment, implementation, and post-deployment evaluation [102]. Where vaccination is implemented, conservation programs should integrate it with biosecurity measures, habitat management [12, 23], and host-genomic risk assessment [26]. At the international level, priority actions should include development of regional genomic surveillance networks informed by phylodynamic evidence [16, 105], improved access to affordable diagnostics for range countries, and CITES-compatible agreements for standardized trade and surveillance data sharing [89].

### Policy recommendations

International trade regulations should recognize BFDV screening as an essential biosecurity component of live psittacine movement rather than an optional administrative requirement. Although CITES provides the principal international framework for regulating trade in threatened species [89], current permit systems do not mandate standardized pathogen screening. A practical regulatory baseline should incorporate pre-export quarantine with multi-matrix qPCR, followed by sequencing or HRM confirmation for positive samples or atypical amplification profiles [17, 36]. For the movement of endangered species, three additional measures should be implemented: environmental surveillance of source aviaries [8], chain-of-custody diagnostic certification linked to trade permits [89], and post-import quarantine before release into breeding or conservation facilities. This integrated approach is supported by evidence of trade-associated BFDV phylodynamics [16, 105] and persistent traceability gaps within online wildlife markets [72].

Concurrently, national recovery programs and conservation translocation frameworks should explicitly incorporate BFDV into wildlife disease risk assessments [102]. Species-recovery plans should include dedicated BFDV components covering the viral status of source populations, biosecurity audits of captive facilities, baseline assessments of local exposure, and environmental monitoring of nest-site contamination [8]. Longitudinal post-release molecular surveillance should also be incorporated into routine disease risk review cycles [102]. Considering recent BFDV detections in wild great green macaws [11] and the limited demographic resilience of the Spix's macaw reintroduction program [12, 73], disease risk assessments should be conducted proactively before translocation or release rather than only after infection has been detected.

### One health linkages

Although BFDV is not currently recognized as a zoonotic pathogen, it aligns with a One Health framework because it intersects with wildlife trade, captive animal welfare, endangered species conservation, and environmental contamination [112]. Within this context, psittacines and selected sympatric bird species may serve as sentinel hosts for monitoring circovirus circulation at wildlife trade, rehabilitation, and conservation interfaces. Reports of BFDV detection in passerines indicate that PCR-positive non-psittacine birds are not uncommon in Australian surveillance studies [14]. Likewise, detections in rainbow bee-eaters and griffon vultures demonstrate that positive findings in coraciiform and raptor species should prompt structured epidemiological investigations rather than immediate assumptions of reservoir status [24, 113].

BFDV should also be interpreted within the broader context of circovirus emergence. PCV2 and PCV3 illustrate how small single-stranded DNA circoviruses can disseminate globally, undergo recombination, and diversify under the selective pressures imposed by livestock movement and host immunity, whereas reports of avian circoviruses suggest that host range is more appropriately viewed as a surveillance continuum than as a fixed biological boundary [39, 85, 86]. One practical extension of this concept would be development of an open-access BFDV sequence database and genomic surveillance network modeled conceptually on Nextstrain and GISAID [109, 110]. At a minimum, deposited records should include complete-genome sequences whenever available, clade assignments, recombination annotations, host species, sample matrix, year and country of collection, wild, captive, or trade origin, diagnostic methodology, viral load proxy, and relevant clinical or conservation metadata. To protect threatened populations, precise geographic coordinates for endangered species should be masked or made available only through controlled-access systems. Such comparative surveillance platforms could distinguish routine BFDV genetic diversity from unusual emergence events without compromising sensitive conservation locations.

### Ethical and capacity considerations

Vaccination of wild or reintroduced psittacine populations requires careful balancing of capture-associated stress against the potential benefits of disease risk reduction. Active intervention is most appropriate for small or vulnerable populations facing substantial disease pressure, particularly where non-invasive surveillance alone is insufficient and formal disease risk analyses support escalation [102]. Candidate vaccines should demonstrate substantially stronger evidence of target-species safety, immunogenicity, and protective efficacy than is currently available from preclinical formulation studies and limited historical challenge experiments [19, 25]. To minimize adverse welfare impacts, field programs should prioritize non-invasive environmental surveillance whenever feasible rather than routine physical capture [8]. Vaccination protocols should also incorporate staged ethical approval, predefined stopping criteria, and adaptive observational study designs.

Successful implementation of BFDV control also depends on strengthening local diagnostic capacity and ensuring sustainable financial support. Strategic investments should prioritize regional reference laboratories, validated portable molecular diagnostic platforms, and subsidized testing for conservation programs and wildlife rehabilitation facilities. Although the BFDV LAMP assay provides an important foundation for low-equipment molecular testing, it should not be regarded as a comprehensive field deployment solution [5]. Wildlife enforcement training should focus on high risk trade pathways where traceability remains weakest [2, 72]. In addition, genomic surveillance initiatives require robust data-governance frameworks that protect sensitive host and pathogen information while facilitating appropriate international data sharing to strengthen global biosecurity [16, 110].

## CONCLUSION

BFDV remains one of the most significant infectious threats to psittacine conservation because of its remarkable genetic diversity, environmental persistence, extensive global dissemination through legal and illegal bird trade, and capacity to establish persistent infections. This review demonstrates that substantial progress has been achieved in understanding BFDV molecular biology, epidemiology, diagnostics, host-pathogen interactions, and emerging vaccine technologies. Advances in whole-genome sequencing, phylodynamic analyses, high resolution molecular diagnostics, and experimental vaccine platforms have substantially improved our understanding of viral evolution and transmission dynamics while providing new opportunities for evidence-based disease surveillance and control.

Despite these advances, several important challenges continue to limit effective BFDV management. Current surveillance systems remain fragmented because of heterogeneous diagnostic methodologies and the absence of standardized reference materials and reporting criteria. Although vaccine development has progressed considerably, protective efficacy across genetically diverse viral lineages and practical field deployment remain unproven. Likewise, uncertainty surrounding subclinical persistence, intermittent viral shedding, environmental contamination, and the epidemiological significance of cross-species detections continues to complicate disease risk assessment and conservation decision-making. These knowledge gaps emphasize the need for harmonized diagnostics, longitudinal surveillance, mechanistic investigations of host-resilience, and coordinated international genomic surveillance.

The resilience framework proposed in this review provides an integrated strategy that links molecular epidemiology, diagnostic harmonization, genomic surveillance, biosecurity, conservation planning, and policy development into a practical decision-support approach. Rather than relying on individual interventions, effective BFDV control will require coordinated implementation of standardized molecular surveillance, evidence-based quarantine protocols, environmental monitoring, risk-informed wildlife trade regulation, and adaptive conservation management. The proposed research prioritization matrix and phased roadmap offer practical guidance for translating current evidence into measurable scientific and policy outcomes while remaining sufficiently flexible for refinement as new data emerge.

Ultimately, sustainable mitigation of BFDV will depend on strengthened international collaboration among researchers, veterinarians, wildlife managers, conservation organizations, diagnostic laboratories, and regulatory authorities. Continued investment in standardized surveillance systems, target-species vaccine evaluation, host-genomic research, accessible diagnostic capacity, and integrated conservation governance will be essential for reducing the global impact of BFDV. Collectively, these efforts will facilitate a transition from reactive disease management toward predictive, evidence-based conservation strategies that enhance the long-term resilience of wild and captive psittacine populations while supporting biodiversity conservation worldwide.

## DATA AVAILABILITY

The data supporting this review are included within the manuscript.

## GENERATIVE AI DECLARATION

During the preparation of this manuscript, the author used Gemini (Google) solely to assist in the graphical design and visual refinement of Figure 3. Artificial intelligence was not used to generate, analyze, or interpret scientific data, formulate scientific conclusions, or draft the manuscript content. All AI-assisted graphical outputs were critically reviewed, modified where necessary, and verified by the author to ensure scientific accuracy, originality, and consistency with the manuscript. The author accepts full responsibility for the integrity and content of this publication.

## AUTHORS’ CONTRIBUTIONS

HG: Conceived and designed the review; performed the literature search, literature screening, evidence synthesis, and manuscript drafting; prepared the figures; supervised the study; and critically reviewed and revised the manuscript. The author has read and approved the final version of the manuscript.
